# Autophagy in the Regulation of Tissue Differentiation and Homeostasis

**DOI:** 10.3389/fcell.2020.602901

**Published:** 2020-12-10

**Authors:** Cristiana Perrotta, Maria Grazia Cattaneo, Raffaella Molteni, Clara De Palma

**Affiliations:** ^1^Department of Biomedical and Clinical Sciences “Luigi Sacco” (DIBIC), Università degli Studi di Milano, Milan, Italy; ^2^Department of Medical Biotechnology and Translational Medicine (BIOMETRA), Università degli Studi di Milano, Milan, Italy

**Keywords:** autophagy, tissue homeostasis, tissue differentiation, tissue remodeling, tissue pathophysiology

## Abstract

Autophagy is a constitutive pathway that allows the lysosomal degradation of damaged components. This conserved process is essential for metabolic plasticity and tissue homeostasis and is crucial for mammalian post-mitotic cells. Autophagy also controls stem cell fate and defective autophagy is involved in many pathophysiological processes. In this review, we focus on established and recent breakthroughs aimed at elucidating the impact of autophagy in differentiation and homeostasis maintenance of endothelium, muscle, immune system, and brain providing a suitable framework of the emerging results and highlighting the pivotal role of autophagic response in tissue functions, stem cell dynamics and differentiation rates.

## Introduction

In the 1990s, the discovery of Atg genes paved the way to the awareness of the role of autophagy in embryonic differentiation and development of both invertebrates and vertebrates (Mizushima and Levine, 2010; Agnello et al., 2015). Actually, autophagy is an evolutionarily conserved process, and the orthologs of most Atg genes, originally discovered in *Saccharomyces cerevisiae* (Ohsumi, 2001), have been isolated and functionally characterized in higher eukaryotes. Genetic knockout of Atg genes has revealed the dependence on autophagy for the formation of spores in yeast (Tsukada and Ohsumi, 1993) and dauer larvae in *Caenorhabditis elegans* (Melendez et al., 2003), and for insect metamorphosis in *Lepidoptera* and *Drosophila melanogaster* (Romanelli et al., 2016). Systemic and tissue-specific knockout of Atg genes in murine models has proven the involvement of autophagy also in mammalian development and differentiation (Mizushima and Levine, 2010).

Autophagy process can be distinguished according to how cargo enters the lysosome compartment. Consistently, three different pathways can be recognized: chaperone-mediated autophagy (CMA), microautophagy and macroautophagy. In CMA, proteins with a specific motif, that are typically subjected to unfolding or denaturation, are recognized by molecular chaperones and directly driven into lysosomes. In microautophagy, cytoplasmic components are directly engulfed into the lysosomal compartment, while in macroautophagy, autophagosomes, characterized by a double membrane structure, surround the cytoplasmic components (Mizushima et al., 2008) and fuse with lysosomes, where their content is degraded. Macroautophagy, commonly and hereafter referred to as autophagy, provides amino acids and energy from the bulk degradation and recycling of intracellular components (Klionsky, 2007).

At first autophagy activation was identified as the response to starvation (Mortimore and Schworer, 1977); currently, we know that autophagy is activated in response to different cellular stressors including exercise, endoplasmic reticulum stress, infection, and hypoxia (Kroemer et al., 2010). Autophagy is a multi-step process with an ordered sequence of events that include induction, nucleation of a phagophore structure, formation and maturation of autophagosome, and finally autophagosome fusion with lysosome to degrade and recycle nutrients (Mizushima, 2007). The proper execution of autophagy relies on the formation of two crucial protein complexes and two sequential conjugation steps. The UNC51-like kinase 1 (ULK1) kinase protein complex is responsible for the initiation step of the process and it is directly regulated by the nutrient-sensing mammalian target of rapamycin (mTOR) that, phosphorylating ULK1, prevents its interaction with the energy-sensing AMP-activated protein kinase (AMPK) and blocks the complex assembling. Moreover, AMPK can directly phosphorylate ULK1 promoting the formation of the complex (Kim et al., 2011) that additionally requires Atg13 phosphorylation and the scaffold protein FAK family kinase interacting protein of 200 kDa (FIP200) resulting in a multi-protein complex composed of ULK1-Atg13-FIP200-ATG101. This accounts for the activation of another multi-protein system, the phosphatidylinositol 3-kinase (PI3K) complex. This complex consists of VPS34, VPS15, beclin-1, Atg14L, and AMBRA1 and is involved in autophagosomes biogenesis (Simonsen and Tooze, 2009). The PI3K complex provides phosphatidylinositol 3-phosphate (PI(3)P) enrichment at specific membrane sites called omegasomes or phagophore assembly site (PAS), that are dynamically connected to the endoplasmic reticulum (ER). Omegasomes are in contact with both conjugation systems and are well-suited for nucleation step, while the connection with ER ensures a good source of the lipids that are used in the conjugation step; moreover, omegasomes are important for recruiting effectors such as Atg18, Atg20, Atg21 (Axe et al., 2008). However, not only ER but other different organelles have been suggested to supply membrane to form phagophores, including plasma membrane, Golgi, mitochondria, and recycling endosomes.

The occurrence of ubiquitin-like conjugation reactions is crucial for elongation and closure of autophagosomes. There are two ubiquitin-like Atg conjugation systems, Atg5–Atg12 and microtubule-associated protein 1 light chain 3 (LC3/Atg8). The conjugation of Atg12 and Atg5 is mediated by Atg7 and Atg10. Next, Atg12-Atg5 associates with Atg16L1 forming a complex that acts as an E3-like ligase. LC3 is first cleaved by Atg4, then in response to autophagy induction is conjugated with phosphatidylethanolamine, by Atg7 together with Atg3 and the complex Atg5–Atg12:Atg16L1. This lipidated form of LC3, also known as LC3II, is incorporated into the autophagosomes during the elongation process (Mehrpour et al., 2010) and it is a common marker of autophagy induction.

Finally, autophagosomes, carrying cytosolic components and dysfunctional organelles, fuse with lysosomes with a mechanism that requires SNARE, Rab and membrane tethering proteins; however, they can also merge with endocytic compartments before reaching the lysosomes. In the lysosomes, the content of autophagosomes is degraded and exported back to the cytosol to fuel new nutrients. Several molecular signals drive and control this complex process including the transcription factors c-Jun N-terminal kinase (JNK), NFKappaB, Hypoxia-Inducible Factor 1(HIF-1), E2F Transcription Factor 1 (E2F1), Forkhead Box proteins (FoxOs) and p53 (Mehrpour et al., 2010) that act at nuclear levels regulating the expression of genes important for autophagy.

Under normal conditions, autophagy enables long-lived protein breakdown, therefore complementing proteasomal activity on short-lived proteins and helps the cell to remove damaged organelles, such as mitochondria. However, autophagy functions can be extended beyond and here we review its essential role in maintaining cell survival and tissue homeostasis under physiological or stressed conditions focusing on how modification of its levels can be useful for tissues adaptations or detrimental, altering their functions.

In this review, we explore autophagy process in endothelium, muscle, immune system, and brain providing a suitable framework of the emerging results and highlighting the pivotal role of autophagic response in tissue differentiation, functions, and remodeling after stimuli.

## Autophagy and Endothelium Homeostasis

The ability of autophagy to drive rapid cellular changes and tissue remodeling in response to environmental and hormonal cues favors the main role of this process in the formation of new blood vessels and vascular homeostasis. A strictly regulated cellular and tissue remodeling is indeed required either for vasculogenesis - that is the differentiation of endothelial precursor cells into endothelial cells (ECs) and *de novo* formation of a primitive vascular network that remodels to acquire tissue- and organ-specific functionality – or angiogenesis, the growth of new blood vessels from pre-existing ones *via* sprouting or intussusception. Specific ECs can also derive from local progenitors in different organs or tissues, further enhancing the complexity and diversity of the endothelial response to injury and regenerative capacity (Marcu et al., 2018). Nonetheless, albeit the phenotypical plasticity characterizing ECs is suggestive of a key role for autophagy in development, differentiation and homeostasis of the endothelium, knowledge about this issue is still sparse and incomplete. Table 1 summarizes the present understanding on the role of autophagy in endothelial behavior, focusing on the genetic or pharmacological approaches currently available to modulate the autophagic process both *in vitro* and *in vivo*. All the data presented in Table 1 are deeply discussed in the sections below.

**TABLE 1:** A summary of the current knowledge on the role of autophagy in the maintenance of endothelial behavior and functions.

*In vitro* genetic or pharmacological modulation	Autophagy	Cell type	Biological effect	References
Atg3 silencing	↓	ECs	Decrease eNOS expression, and increase ROS and inflammation	Bharath et al., 2014
Resveratrol	↑	ECs	Reduce vascular inflammation	Chen et al., 2013
Rapamycin	↑	ECs	Increase hemodynamic shear stress-induced eNOS expression	Guo et al., 2014
Atg5 overexpression	↑	ECs	Increase sprouting	Du et al., 2012
Atg5 silencing or 3-MA treatment	↓	ECs	Reduce sprouting	Du et al., 2012
ULK1 silencing	↓	ECs	Reduce VEGF- or AGGF1-induced sprouting	Lu Q. L. et al., 2016; Spengler et al., 2020
Beclin-1 silencing	↓	ECs	Reduce EC-haematopoiesis supporting ability	Lyu et al., 2020
3-MA	↓	MSCs	Favor stemness maintenance	Chang et al., 2015
Rapamycin	↑	MSCs	Exert a protective effect against senescence	Zhang et al., 2020
			Increase VEGF secretion and angiogenesis	An et al., 2018
Beclin-1 silencing	↓	MSCs	Reduce VEGF-proangiogenic effect	An et al., 2018
***In vivo* genetic or**				
***pharmacological modulation***				
EC-selective Atg7 knock out	↓	Mice	Reduce postnatal microvessel brain density	Zhuang et al., 2017
		ApoE–/– mice	Favor development of atherosclerotic lesions	Torisu et al., 2016
EC-selective Atg5 knock out or chloroquine	↓	Mice	No effect in retinal vasculogenesis	Sprott et al., 2019; Zhao et al., 2019
		Mice	Larger atherosclerotic lesions in area exposed to shear stress	Vion et al., 2017
Chloroquine	↓	Melanoma mouse model	Normalize tumoral vessels	Maes et al., 2014
Preconditioning hypoxia or atorvastatin	↑	Engrafted MSCs	Promote survival	Zhang Y. et al., 2012

### Autophagy and Vasculogenesis

The formation of blood vessels *via* vasculogenesis is critical for the development of any new tissue during embryogenesis (Sweeney and Foldes, 2018). The early identifiable vessels arise in the yolk sac where primitive ECs - derived from the mesodermal layer of the embryo and expressing endothelial markers including Vascular Endothelial Growth Factor Receptor (VEGFR), VE-cadherin and CD31 (Breier et al., 1996) – aggregate to form blood islands, and then migrate to the fetus where vascular networks are formed. The expression of Atg7, Atg8, and beclin-1 has been described in the vascular plexus of the chick yolk sac and chorioallantoic membrane, and developing chick embryos reveal a hemorrhage phenotype – due to failure of capillary endothelium - when exposed to either inducer or inhibitor of autophagy (Lu W. H. et al., 2016). A reduced brain microvessel density has been reported in transgenic mice with endothelial-selective knockout of Atg7 (Zhuang et al., 2017). This inhibitory effect on cerebral vascular density seems to mostly rely, however, on defective postnatal angiogenesis rather than embryonal vasculogenesis (Zhuang et al., 2017). More recently, it has been observed that the physiological development of retinal vasculature is unaffected by endothelium-specific Atg5 deletion (Sprott et al., 2019) or by pharmacological inhibition of autophagy (Zhao et al., 2019). Therefore, the role of autophagy in vasculogenesis is still obscure, and further studies will be required to fully understand its impact on embryonic vascularization.

### Autophagy and Angiogenesis

Blood vessels carry oxygen, nutrients, and immune cells to all the body’s tissues and are crucial for tissue growth and physiology also in adult vertebrates. In healthy organisms, ECs lining blood vessels are quiescent but still retain the ability to respond to micro-environmental changes or pro-angiogenic signals to form new blood vessels. Inadequate vessel formation and maintenance as well as abnormal vascular remodeling underlie various diseases including myocardial infarct, stroke, cancer, and inflammatory disorders.

Several findings in cellular and animal models suggest that autophagy may critically regulate vascular sprouting. Overexpression of the Atg5 gene induces *in vitro* tubulogenesis whereas Atg5 silencing or drug-induced inhibition of autophagy suppress sprouting (Du et al., 2012). Functional autophagy is required for *in vitro* angiogenesis induced by VEGF (Spengler et al., 2020) or by the angiogenic factor AGGF1 (Lu Q. L. et al., 2016), even if different molecular pathways are engaged by the two factors to activate autophagy. Notably, the expression of AGGF1 is induced in ischemic myocardium, and AGGF1 knockout mice show reduced autophagy and angiogenesis and larger damaged areas after myocardial infarction (Lu Q. L. et al., 2016). Angiogenic factors can therefore induce autophagy, and autophagy, acting upstream of angiogenesis, is essential for neovascularization. Angiogenesis also relies on EC autophagy in a rat model of burn wound (Liang et al., 2018). Consequently, induction of autophagy might provide a novel approach to boost the efficacy of therapeutic angiogenesis in ischemic diseases and tissue regeneration.

### Autophagy and the Angiogenic Potential of Stem Cells

Different types of stem cells (SCs), especially mesenchymal stem/stromal cells (MSCs), have been extensively investigated for proangiogenic cell therapy in hypo-vascular injuries, such as peripheral artery disease, myocardial infarction, and stroke (Bronckaers et al., 2014). A crucial issue in SC biology concerns the balance between stemness and differentiation, and accumulating evidence suggests that autophagy is critical for the maintenance of self-renewal properties of long-lived SCs and for the differentiation of either embryonic or adult SCs (Ho et al., 2017; Boya et al., 2018; Chen X. H. et al., 2018; Chang, 2020). Likewise, stemness and differentiation are regulated by autophagy in MSCs (Sbrana et al., 2016; Jakovljevic et al., 2018) with the autophagic activity of old bone marrow-derived MSCs reduced in comparison with young MSCs (Ma et al., 2018). However, the relationship between autophagy and senescence has not been totally elucidated in MSCs (Rastaldo et al., 2020). The pharmacological inhibition of autophagy by 3-methyladenine (3-MA) maintains stemness in high glucose-treated MSCs (Chang et al., 2015). Accordingly, upregulation of autophagy in hyperglycemic conditions correlates with ROS accumulation and premature senescence (Chang et al., 2015), and increased levels of autophagy-related genes have been found in senescent MSCs (Fafian-Labora et al., 2019). At variance, a protective role for rapamycin-induced autophagy accompanied by a decrease in ROS production has been shown in a D-galactose-mediated model of MSC aging (Zhang et al., 2020), and the autophagic flux is compromised in other models of acute senescence, suggesting that functional autophagy may be required to counteract detrimental pathways whereas its negative modulation favors the establishment of cellular aging (Song et al., 2014; Capasso et al., 2015). The protective effect of autophagy may be decisive when MSCs are engrafted in regions characterized by a severe oxidative environment, such as infarcted hearts, where most of transplanted MSCs die in a few days due to hypoxic stress-induced apoptosis (Miao et al., 2017). Notably, induction of autophagy by preconditioning MSCs with brief hypoxia prior to transplantation in ischemic myocardium promotes their survival (Zhang Q. et al., 2012). Also drugs that activate autophagy, such as atorvastatin, decrease hypoxia-induced apoptosis and enhance survival of transplanted MSCs (Zhang Q. et al., 2012). Remarkably, hypoxic priming of MSCs before transplantation improves viability and pro-angiogenic potential of engrafted MSCs also in the treatment of diabetic complications such as lower limb ischemia (Qadura et al., 2018). Collectively, all these studies suggest that modulation of autophagy may be a general approach to protect MSCs from external/internal stressors, thus enhancing their survival in engrafted tissues, and finally their regenerative and therapeutic potential (Ceccariglia et al., 2020). The pro-angiogenic potential of MSCs is also controlled by their paracrine activities that are due to all the factors released from cells, collectively defined as secretome (Maacha et al., 2020). Rapamycin-induced autophagy increases VEGF secretion from MSCs and accelerates regeneration in a murine model of wound healing whereas beclin-1 silencing blunts VEGF-mediated MSC pro-angiogenic effects (An et al., 2018). Consequently, the ability of autophagy to influence secretome may represent a further level of control of the regenerative/therapeutic potential of MSCs. It is therefore possible to summarize that autophagy favors revascularization of ischemic areas either by directly promoting angiogenesis or by supporting the angiogenic potential of MSCs through multiple mechanisms.

Endothelial cells are also essential elements of the hematopoietic SC (HSC) niche by providing critical signals to support blood cell production in the bone marrow (Mendelson and Frenette, 2014). Again, autophagy has been proposed as a key regulator of this process because beclin-1 knockdown reduces the hematopoiesis-supporting ability of ECs that can be restored by beclin-1 upregulation (Lyu et al., 2020). Notably, a prospective case-control study illustrates that defective autophagy of ECs in the HSC niche may be involved in the post-allotransplant pancytopenia characteristic of poor graft function (PGF), suggesting the possibility of treating PGF patients with drugs able to promote autophagy (for example, rapamycin).

### Autophagy and the Physiopathology of ECs

At variance with post-ischemic angiogenesis, that restores blood flow in hypo-vascularized areas, overgrowth of abnormal vessels results in pathological angiogenesis and underlies various diseases including retinopathies, cancer, and inflammatory disorders. Endothelium-specific deletion of Atg5 reduces pathological neo-vascularization in a mouse model of retinopathy (Sprott et al., 2019), and the retinal vascular hyper-sprouting phenotype induced by PKA deficiency is partially rescued by inhibition of autophagy or endothelium-specific Atg5 deletion (Zhao et al., 2019). Outstanding, defective autophagy does not harm physiological development of retinal vasculature in both the models. Thus, blocking autophagy may be useful to selectively target pathological neo-vascularization, at the retina as well as in tumors, where the formation of new blood vessels is essential for cancer progression. Tumor-associated blood vessels are highly permeable and unstable, and these anomalies promote a stressful environment – characterized by hypoxia, nutrient deprivation and inflammation - that results in enhanced autophagy and resistance to hypoxia-induced cell death in tumoral ECs compared to normal ECs (Filippi et al., 2018). Hypoxia drives not only autophagy but also angiogenesis *via* increased expression of VEGF due to stabilization and activation of the hypoxia-inducible factor HIF. Tumors in hemizygous beclin-1 mice show higher angiogenic potential under hypoxia in comparison to wild type animals (Lee et al., 2011), thus supporting the view that hypoxia-induced autophagy may restrain pathological angiogenesis in the tumoral microenvironment.

The aberrant structure and function of peritumoral vessels supports malignancy by establishing an abnormal tumoral microenvironment that facilitates disease progression and reduces the efficacy of antiblastic therapies. Hypoxia makes cancer cells more aggressive and favors angiogenesis and immunosuppression, while leaky vessels give tumor cells passage to metastasize. It has been hypothesized that normalization of tumor vessels can improve their functions by relieving microenvironmental hypoxia and improving delivery and outcome of anti-cancer drugs (Martin et al., 2019). Remarkably, the autophagy blocker chloroquine (CQ) normalizes tumor vessel structure and function and increases perfusion in a mouse model of melanoma (Maes et al., 2014). Through the alteration of acidic pH in endothelial late endosomes or lysosomes, CQ disrupts endosomal and autophagic cargo degradation, followed by the activation of Notch signaling and negative regulation of tip cell during sprouting angiogenesis. Notch signaling has also been implicated in vessel stability by regulating the function of vascular mural cells (Kofler et al., 2011). In addition, the CQ-mediated reduction of tumor hypoxia favors the establishment of a less pro-angiogenic microenvironment (Maes et al., 2014). Besides autophagy, CQ improves the immunosuppressive function of the tumoral micro-environment by enhancing the switch of tumor-associated macrophages from tumor-promoting M2 to tumor-killing M1 phenotype (Chen D. et al., 2018) – a mechanism that has been related to normalization of tumor vascular network (Jarosz-Biej et al., 2018). Notably, pleiotropic effects of CQ on tumoral vessels are not phenocopied by loss of Atg5, and more generally, results obtained by genetically interfering with the expression of autophagy-related genes do not routinely overlapped to the effects of pharmacological modulation of the endo-lysosomal system (Schaaf et al., 2019). Thus, it is possible to speculate that autophagy might be modulated by complementary approaches – acting at different steps of the autophagic process – to control pathological angiogenesis. It is, however, crucial to further study whether and how these mechanisms intersect not only at the level of ECs but also in other cell types belonging to the tumoral microenvironment.

A crucial role for autophagy has also been proven in terminally differentiated cells, such as ECs lining blood vessels, where a constant renewal of cytoplasmic contents and organelles is essential for the maintenance of homeostasis and cellular health (Mizushima and Komatsu, 2011). An impairment in EC functions – the so-called endothelial dysfunction (ED) - is associated with all the common cardiovascular risk factors and stressors, such as for example aging, smoking, hypertension, diabetes, and low physical activity. ED is triggered by a loss in the endothelial Nitric Oxide Synthase (eNOS) enzymatic activity with a consequent decrease in nitric oxide (NO) availability and accumulation of damaging ROS (Li et al., 2013). Persistent oxidative stress alters mitochondrial structure and function but the efficient degradation and recycling of damaged organelles *via* autophagy/mitophagy results in cellular survival and homeostasis. At variance, the partial/incomplete degradation of mitochondria due to ineffective autophagy can cause a further increase in oxidative stress, and finally cell death (Yan and Finkel, 2017). Consequently, the activation of autophagy in response to oxidative stress, but also to other vascular stressors such as high glucose, oxidized low-density lipoproteins or advanced glycation end products, exerts a protective effect on ECs, and any alteration in the autophagic flux can elicit detrimental effects (Chen F. et al., 2014; Torisu et al., 2016; Yan and Finkel, 2017). In addition, some vasculo-protective compounds have been shown to stimulate autophagy, thereby reinforcing EC resistance to cellular stress (Chen et al., 2013; Kim H. S. et al., 2013). Hemodynamic shear stress, the mechanical force generated by blood flow on vascular ECs, is essential for endothelial homeostasis under physiological conditions (Hsieh et al., 2014). Pulsatile laminar shear stress upregulates the expression and activity of eNOS, and pretreatment with the autophagy activator rapamycin further enhances shear stress-induced eNOS expression (Guo et al., 2014). Likewise, either autophagy markers or eNOS activity increase in response to physiological levels of laminar flow, and silencing of the Atg3 protein impairs eNOS function and generates ROS and inflammatory cytokines (Bharath et al., 2014). Thus, autophagy sustains pleiotropic functions of NO in the control of barrier integrity, vessel dilation, leukocyte adhesion, platelet aggregation, and finally angiogenesis, by favoring its formation in response to physiological shear stress. In contrast, ECs exposed to low levels of shear stress are characterized by inefficient autophagy and by a proinflammatory, apoptotic, and senescent phenotype (Vion et al., 2017). Similarly, perturbed/unstable flow impairs p62-mediated clearance of autophagosomes and promotes ED (Vion et al., 2017).

The autophagy process has typically been studied in cultured ECs, which are proliferative cells at variance with quiescent cells within the vasculature. However, a protective role for autophagy has also been confirmed *in vivo* where the genetic inactivation of the Atg7 gene favors the development of atherosclerotic lesions in murine models (Torisu et al., 2016). Likewise, atherosclerosis-prone mice bearing an endothelial-specific deletion of Atg5 develop larger atherosclerotic lesions, specifically in areas exposed to high shear stress (Vion et al., 2017), that commonly remain lesion-free since atherosclerosis favorably develops at arterial bifurcations and at the inner part of curvatures where blood flow is low or disturbed (Hsieh et al., 2014). The translational relevance of these findings has been confirmed by an increase in autophagic markers and NO production in human ECs collected from the radial artery of subjects after dynamic handgrip exercises (Park et al., 2019). At variance, reduction in autophagic markers and impaired eNOS activation have been observed in peripheral venous ECs from diabetic patients showing a lower brachial artery flow-mediated dilation suggestive of ED (Fetterman et al., 2016). It can therefore be assumed that: (i) a defect in endothelial autophagy enhances pro-atherogenic responses; (ii) the activation of the autophagic flux by adequate shear stress acts as athero-protective mechanism. Enhancing endothelial autophagy within the vasculature might therefore represent a novel and attractive target for the prevention and/or treatment of atherosclerosis and cardiovascular disease (Hughes et al., 2020).

## Autophagy and Skeletal Muscle Homeostasis

Skeletal muscle is a plastic tissue that is continuously adapting and changing to physical and metabolic demands. To sustain the increased energy needs or cope with catabolic conditions, skeletal muscle can mobilize proteins, reorganize organelles networks, and change the nuclei setting. Autophagy is the major reservoir of energy and the mouse model expressing fluorescent LC3 shows that muscles have one of the highest rates of autophagy during fasting (Mizushima et al., 2004). Basal autophagy is regulated by metabolic properties of muscle, indeed a negative correlation between fiber oxidative capacity and autophagy flux exists and both basal and stimulated autophagy are greater in glycolytic muscles as compared with the oxidative ones (Mofarrahi et al., 2013). These differences are associated with different activation of the AMPK pathway and inhibition of the AKT and mTOR signaling (Mofarrahi et al., 2013) that are more evident in low oxidative muscles.

### Autophagy and Muscle Mass Maintenance

Autophagy maintains healthy muscle homeostasis and physiology (Masiero et al., 2009), exerting a beneficial role in controlling muscle mass. Muscle-specific deficiencies in essential autophagy factors such as Atg5, Atg7, VPS15, ULK1, AMPK, and mTOR result in abnormal mitochondrial morphology, oxidative stress, sarcomere alterations, accumulation of ubiquitylated products, and induction of unfolded protein response that can contribute to great muscle loss and weakness leading to myofibers degeneration (Raben et al., 2008; Masiero et al., 2009; Castets et al., 2013; Nemazanyy et al., 2013; Bujak et al., 2015; Fuqua et al., 2019). Similarly, AMBRA1 deletion results in a severe myopathy with loss of muscle fibers, sarcomere disorganization, and mitochondria alterations (Skobo et al., 2014). AMBRA1 interacts with Tripartite motif-containing 32 (TRIM32) (Di Rienzo et al., 2019) that is crucial as well, and indeed its deletion causes myopathy with neurological complications, both superimposable with the phenotype observed in patients with limb-girdle muscular dystrophy 2H (LGMD2H) (Saccone et al., 2008). Recently, the autophagic lysosome reformation (ALR) pathway has also emerged as an important regulator for muscle homeostasis. Hence, the deletion of the phosphatase regulating the PI(4,5)P2 to PI(4)P conversion, i.e., the inositol polyphosphate 5-phosphatase (INPP5K), causes a progressive muscle disease associated with lysosomes depletion and autophagy impairment (McGrath et al., 2020). Whether in other systems the Transcription Factor EB (TFEB) is able to restore lysosomal homeostasis and autophagy (Spampanato et al., 2013), in this model TFEB-dependent lysosomal biogenesis is not sufficient to compensate the defective ALR, indicating its fundamental role in replenishing lysosomes during autophagy.

Interestingly, mild perturbation of autophagy does not cause a severe muscular phenotype. Muscle appears normal despite a slight, but non-significant, reduction of its mass and a small decrease in fibers size regardless of type (Paolini et al., 2018), highlighting the differences between models in which autophagy is completely blocked or only mild attenuated. In this model, muscle loss is evident after a starvation period suggesting that the absence of inducible autophagic response speeds up the process reducing protein synthesis and increasing ubiquitylated products and confirms how a normal autophagic process is essential for maintaining muscle mass (Paolini et al., 2018).

The preservation of muscle mass could be achieved by the maintenance of Sestrins levels, which appear strongly downregulated in the atrophic muscle (Segales et al., 2020) and aged-subjects (Zeng et al., 2018). Sestrins is able to enhance autophagy preserving organelles quality, and in turn muscle mass, through a double mechanism: it inhibits FoxO-dependent expression of atrogenes by increasing Akt, and sustains autophagy, by activating AMPK and blocking mTORC1 (Segales et al., 2020). Other factors are emerging as important for controlling and averting atrophy; especially, recent evidence points out that TRIM32 is required for autophagy induction in atrophic conditions promoting ULK1 activity. This improves the functional maintenance of muscle cells *via* reduction of ROS production and induction of muscle ring finger-1 (MuRF1) (Di Rienzo et al., 2019).

Of note, whether the absence of autophagy is harmful, its excessive induction is detrimental too. For instance, the upregulation of FoxO3 transcription factor enhances autophagy and is enough to induce muscle fibers atrophy (Mammucari et al., 2007). Similarly, mice lacking the nutrient-deprivation autophagy factor-1 (NAF-1), an endoplasmic reticulum (ER) protein required for blocking beclin-dependent autophagy, display muscle weakness accompanied by increased autophagy (Chang et al., 2012). Consistently, a high autophagy rate exacerbates muscle atrophy induced by several conditions. The lesson from these results is complex, indicating a dual role for autophagy and suggesting that a proper autophagic process is crucial for muscle homeostasis.

### Autophagy and Exercise

Autophagy is also important for skeletal muscle remodeling after stimuli like contraction/exercise. Exercise can help to improve muscle quality in old and frail people (Cadore et al., 2014). Consistently, a recent study sheds light on the effects of acute and chronic exercise on autophagy in frail elderly subjects. The authors establish that autophagy is activated after a session of strength training in unexercised subjects. By contrast, in exercised subjects performing the same training, this response is completely blunted, suggesting that the results of this physical activity depend on the subjects’ training status (Aas et al., 2020). Similarly, resistance exercise enhances autophagy in untrained young men, but it is absent in aged-subjects (Hentila et al., 2018). This last result partially contrasts the work of Aas et al. (2020), suggesting that the autophagic response to strength training needs to be further investigated.

Defective stimulation of basal autophagy impairs metabolic adaptations induced by exercise training, such as mitochondrial biogenesis and angiogenesis, without interfering with the fiber type switch distinctive of contractile adaptations (Lira et al., 2013). This confirms the existence of a link between oxidative phenotype and basal autophagy flux, as well as demonstrates that chronic contractile activity is not sufficient to affect basal autophagy flux (Lira et al., 2013).

Hence, exercise is considered a stimulus that induces autophagy *in vivo* (Grumati et al., 2011; He et al., 2012) and a described mechanism involves the disruption of the BCL2–beclin-1 complex (He et al., 2012). Knock-in mutation in BLC2 phosphorylation site generates mice with normal basal autophagy but defective stimulus-induced autophagy. These mice display normal cardiac and skeletal muscle features at baseline; conversely, they show impaired exercise-enhanced insulin sensitivity and fail to exhibit increased plasma membrane GLUT4 localization, both dictated by low AMPK activation (He et al., 2012). These mice have a much worse running performance and are not protected against high fat diet-induced glucose intolerance (He et al., 2012). However, another study reports exactly opposite results demonstrating that muscle-specific autophagy knockout mice have an improved metabolic profile, including glucose homeostasis, associated with the release of the mitokine FGF21 triggered by dysfunctional mitochondria (Kim K. H. et al., 2013). Consistently, in the same muscle-specific inducible model, the inhibition of autophagy immediately before training does not impact on physical performance, glucose homeostasis, or PRKAA1/AMPK signaling. By contrast, autophagy accounts for the preservation of mitochondrial function during muscle contraction, revealing its important role for muscle injury repair (Lo Verso et al., 2014). Besides, these data uncover the differences between a general inhibition of autophagy with respect to tissue-specific blocking, suggesting a potential cell-autonomous regulation.

### Autophagy and Muscle Regeneration

The finding that autophagy is important in muscle repair has been deeply investigated. The regenerative capacity of muscle is a crucial process that allows the recovery after damage and requires the effort of different cell types.

Autophagy is rapidly induced at the onset of regeneration process showing a role in sarcomeric disassembly but not in nutrient recycling. Autophagy inhibition causes the accumulation of sarcomeric remnants and prevents the proper organization of sarcomere during regeneration (Saera-Vila et al., 2016).

In case of injury, autophagy is required to maintain sarcolemma integrity, as demonstrated in mice with decreased Atg16 function (Atg16L1 mice) and exposed to cardiotoxin (CTX) damage. In absence of autophagy and with low CTX levels, fibers become leaky and are infiltrated by circulating immunoglobulins, but are able to buffer calcium rise preventing their necrosis and death. Otherwise, whether the local concentration of CTX is high, fibers cannot buffer calcium and die; overall, these effects result in attenuated muscle regeneration in Atg16L1 mice (Paolini et al., 2018).

Autophagy can also contribute to mitochondrial regeneration during muscle repair and chronic treatment with an autophagy inhibitor negatively affects mitochondrial activity and recovery after injury (Nichenko et al., 2016). Similarly, the muscle-specific deletion of the ULK1 gene, which is essential for mitophagy (mitochondrial-specific autophagy), causes impaired functional recovery of muscle associated with altered respiratory complexes levels (Call et al., 2017). Specifically, ULK1 is important for the reorganization of the mitochondrial network after damage and this event is crucial for the optimal recovery of muscle (Call et al., 2017).

A role for autophagy in muscle regeneration can be also drawn from the recent discoveries in satellite cells that are functional adult stem cells with the ability to proliferate and undergo myogenesis. Autophagy is essential for satellite cells to come out of their quiescent state, indeed this activation is a metabolic demanding process and autophagy can provide the required nutrients favoring the transition between quiescent to the activated state (Tang and Rando, 2014). In agreement, committed myogenic progenitors are mostly positive for LC3 in contrast to the self-renewal population, suggesting that the presence of LC3 positive satellite cells overlaps with a strong regenerative response after injury (Fiacco et al., 2016).

Autophagy is also important to control cell senescence and its decline is responsible for the rapid entry of satellite cells into senescence accounting for the exhaustion of stem cells pool and defective muscle regeneration. Physiologically, this occurs with aging: indeed, old satellite cells show autophagy decline and are more prone to senescence (Garcia-Prat et al., 2016). The link between autophagy and muscle cell senescence is challenging and it has been further expanded. Stimuli that strongly upregulate autophagy, such as repeated amino acids and serum withdrawal, do not trigger senescence features. Conversely, senescence-associated with mild toxic stress is limited in Atg7-deficient cells for increasing cell death (Bloemberg and Quadrilatero, 2020). This suggests that massive induction of autophagy prevents senescence, whereas autophagy-defective myoblasts do not develop senescence, but die (Bloemberg and Quadrilatero, 2020).

Besides, autophagy controls the effectiveness of GH-IGF1 axis in muscle affecting the proliferative capacity and the differentiation potential of satellite cells and influencing muscle development, with severe post-natal muscle growth retard in condition of autophagy disruption (Zecchini et al., 2019).

The exact role of autophagy in muscle regeneration is complex considering the different cell types involved in the process. However, the induction of autophagy seems to be beneficial for repairing after damage as well as for exercise adaptations.

### Autophagy and Muscle Disorders

Another proof of concept of the role of autophagy in muscle homeostasis comes from the evidence that many pathophysiological conditions of muscle are associated with disrupted autophagic process including muscular dystrophies (Grumati et al., 2010; De Palma et al., 2012, 2014; Fiacco et al., 2016), type II diabetes mellitus and insulin resistance (He et al., 2012), sarcopenia (Fan et al., 2016) and cancer cachexia (de Castro et al., 2019). Moreover, genetic defects affecting each phase of autophagy underlie skeletal muscle illness and the severity of the phenotype depends on whether the mutation disrupts basal or inducible autophagy (Jokl and Blanco, 2016). Interestingly, upregulation as well as downregulation of autophagy, are associated with muscle disorders, confirming the importance of a proper autophagic flux. For instance, in Pompe disease, an excessive autophagic buildup occurs and compromises enzyme replacement therapy (Fukuda et al., 2006; Raben et al., 2008).

In myotonic dystrophy type 1 (DM1), which affects adults causing atrophy and myotonia, autophagy levels are enhanced (Bargiela et al., 2015) and counteracting autophagy by mTOR stimulation improves satellite cells proliferation (Song et al., 2020).

Conversely, in the Vici syndrome, the causative genetic mutation leads to an autophagic block resulting in a clearance defect with accumulation of autolysosomes (Cullup et al., 2013). Autophagy signaling is also impaired in muscular dystrophies as shown in dystrophin or collagen VI deficient muscles and the forced reactivation of the process improves both dystrophic phenotypes (Grumati et al., 2010; De Palma et al., 2012; Fiacco et al., 2016). Specifically, in collagen VI deficient patients a dietary approach for reactivating autophagy has been attempted in a pilot clinical trial, obtaining beneficial effects (Castagnaro et al., 2016). Similarly, the administration of nutraceutical compounds, such as pterostilbene in collagen 6 null mice promotes a proper autophagic flux exerting favorable outcomes on muscle (Metti et al., 2020).

Defective autophagy is also detected in LGDM2H due to impaired interaction between mutated TRIM32 and ULK1 which results in the accumulation of autophagy cargo receptors, ROS production, and MuRF1 upregulation (Di Rienzo et al., 2019). The efficient reactivation of autophagy is also useful in Emery–Dreifuss muscular dystrophy (Ramos et al., 2012); by contrast, in laminin alpha2 chain-deficient muscle autophagy is upregulated and its inhibition has beneficial effects on dystrophic phenotype (Carmignac et al., 2011). In this regard, many other examples indicate that muscle is particularly susceptible to autophagy dysregulation (Dobrowolny et al., 2008; Sarparanta et al., 2012; Fetalvero et al., 2013). Therefore, undoubtedly, autophagy is a key process whose alteration affects development, homeostasis and remodeling of skeletal muscle and whose normalization is essential for the physiology of skeletal muscle as well as for ameliorating muscular diseases, as summarized in Table 2.

**TABLE 2:** A summary of the current knowledge on the role of autophagy in the maintenance of muscle behavior and functions.

*In vitro* genetic or pharmacological modulation	Autophagy	Model	Biological effect	References
3-MA or Chloroquine Atg5 or Atg7 silencing	↓	SC	Inhibition of SC activation	Tang and Rando, 2014
***In vivo* genetic or**				
***pharmacological modulation***				
Muscle-specific knock out models for: Atg5-Atg7-VPS15-ULK1-AMPK-mTOR-AMBRA1	↓	Mice	Mitochondria dysfunctions, sarcomere alterations, muscle loss, weakness, and myofibers degeneration.	Raben et al., 2008; Masiero et al., 2009; Castets et al., 2013; Nemazanyy et al., 2013; Skobo et al., 2014; Bujak et al., 2015; Fuqua et al., 2019
Muscle-specific TRIM32 knockout	↓	Mice	Myopathy with neurological alterations. Mutations in TRIM32 are responsible for LGMD2H	Saccone et al., 2008
Muscle-specific INPP5K knockout	↓	Mice	INPP5K deletion causes muscular dystrophy	McGrath et al., 2020
Muscle-specific FoxO3 upregulation	↑	Mice	Muscle atrophy	Mammucari et al., 2007
Muscle-specific NAF-1 knockout	↑	Mice	Muscle weakness	Chang et al., 2012
Muscle-specific Atg16L1	↓ (mild)	Mice	Impaired regeneration after injury	Paolini et al., 2018
Muscle-specific UlK1 knockout	↓ (mitophagy)	Mice	Impaired regeneration after injury	Call et al., 2017
Muscle stem cell-specific Atg7 knockout	↓	Mice	Impairment of GH-IGF1 axis and severe muscle growth retard. Altered proliferation and differentiation of muscle stem cells	Zecchini et al., 2019
Atg6 knockout	Defective stimulus- induced autophagy	Mice	Defective adaptations to endurance exercise training	Lira et al., 2013
Knock-in mutations in BCL2 phosphorylation sites (Bcl2^AAA^)	Defective stimulus- induced autophagy	Mice	Decreased performance and altered glucose metabolism during acute exercise. Impaired exercise-mediated protection against high-fat-diet-induced glucose intolerance	He et al., 2012
3-MA	↓	Mice	Impaired mitochondrial activity and muscle regeneration after injury	Nichenko et al., 2016
Low protein diet	↑	COLL6 deficient patients mdx mice	Autophagy recovery and amelioration of dystrophic phenotype. Autophagy recovery and amelioration of dystrophic phenotype	De Palma et al., 2012; Castagnaro et al., 2016
Pterostilbene	↑	COLL6 knockout mice	Autophagy recovery and amelioration of dystrophic phenotype	Metti et al., 2020

## Autophagy and Immune System Homeostasis

Autophagy is an essential process in the regulation of homeostasis of immune cells involved in innate and adaptive immune responses, such as monocytes, macrophages, dendritic cells (DCs), T and B lymphocytes, by influencing their proliferation, differentiation, activation, survival and cytokine release (Harris, 2011).

Monocytes and macrophages are pivotal effectors and regulators of the innate immune response (Italiani and Boraschi, 2014). DCs, as antigen-presenting cells (APCs), trigger and modulate the activation of naive T cells, and have a central role in the development and maintenance of immune tolerance (Patente et al., 2018). T cells have diverse jobs: (i) they activate other immune cells (T helper cells) (Glimcher and Murphy, 2000); (ii) they detect and destroy infected and tumor cells (cytotoxic T cells) (Andersen et al., 2006); (iii) they can “remember” a previous encounter with a specific microbe and start a quick response upon pathogen re-exposure (memory T cells) (Omilusik and Goldrath, 2017); (iv) they can regulate or suppress other cells in the immune system (regulatory T cells: Treg) (Vignali et al., 2008). B cells are mainly responsible for mediating the production of antigen-specific antibodies directed against invasive pathogens (Eibel et al., 2014). Autophagy has been demonstrated to be central in all these cells, as it modulates cell signaling and metabolism, antigen presentation, proteostasis, mitochondrial function and reactive oxygen species (ROS) production and ER stress (Rathmell, 2012; Gkikas et al., 2018; Smith, 2018; Valecka et al., 2018; Macian, 2019). An overview of the effects of autophagy changes on immune cells is reported in Table 3 and discussed in the sections below.

**TABLE 3:** A summary of the current knowledge on the role of autophagy in the maintenance of immune cells behavior and functions.

*In vitro* genetic or pharmacological modulation	Autophagy	Model	Biological effect	References
3-MA or chloroquine	↓	Monocytes	Promotion of apoptosis, inhibition of differentiation and of cytokine release	Zhang Y. et al., 2012
Chloroquine	↓	Macrophages	M2 repolarization to M1	Guo et al., 2019
3-MA or cyclosporine A	↓	Macrophages	M1 polarization	Esteban-Martinez et al., 2017
3-MA	↓	Macrophages	Inhibition of M2 polarization	Bo et al., 2020
3-MA or beclin-1 and Atg7 silencing	↓	T cells	Resistance to cell death of Th2 cells	Li et al., 2006
Beclin-1 silencing	↓	ECs	Reduce EC-haematopoiesis supporting ability	Lyu et al., 2020
***In vivo* genetic or**				
***pharmacological modulation***				
Atg5 knock out	↓	Macrophages	Increase of M1 marker expression and cytokine release	Liu et al., 2015
3-MA	↓	Macrophages	Inhibition of M2 polarization	Bo et al., 2020
Atg16L1 knock out	↓	Macrophages	Increase of secretion of IL-1β and IL-18	Saitoh et al., 2008
Beclin-1 knock out or LC3 knock out	↓	Macrophages	Increase of secretion of IL-1β and IL-18	Nakahira et al., 2011
Atg16L1 knock out	↓	Dendritic cells	Boost of the immunostimulatory capability of murine mature DCs	Hubbard-Lucey et al., 2014
Atg5 knock out	↓	T cells	Inhibition of cell survival and proliferation	Pua et al., 2007
Beclin-1 knock out	↓	T cells	Increase of cell death	Kovacs et al., 2012
Plasma cells Atg5 selective knock out	↓	B cells	Plasma cells maintenance and humoral immunity	Pengo et al., 2013
Plasma cells Atg5 selective knock out	↓	B cells	B cell receptor trafficking	Arbogast et al., 2019

### Autophagy and Macrophages

Autophagy plays a key role in different stages of macrophage life, including differentiation from monocytes and polarization into pro-inflammatory and anti-inflammatory and tissue repairing macrophages (i.e., M1 and M2 cells) (Vergadi et al., 2017). Monocytes originate in the bone marrow from a myeloid progenitor and then they are released into the peripheral blood. In response to inflammation, monocytes migrate into tissues and can differentiate into macrophages or die by caspase-dependent apoptosis. Both the cytokines colony-stimulating factor 1 and 2 (CSF1 – CSF2) are able to promote monocyte survival and differentiation into macrophages via autophagy (Jacquel et al., 2012; Zhang Y. et al., 2012). CSF1 contributes to increased induction of autophagy by activating the ULK1 pathway (Jacquel et al., 2012; Obba et al., 2015), while CSF2 acts through JNK/beclin-1 and the block of Atg5 cleavage (Zhang Y. et al., 2012). Of note, the pharmacological blockade of autophagy in CSF2 treated monocytes by 3-MA and CQ leads to the promotion of apoptosis and the inhibition of differentiation and cytokine release, thus corroborating the role of autophagy in these events (Zhang Y. et al., 2012).

To date, it is clear that autophagy is also fundamental in macrophage polarization, i.e., the process by which macrophages develop a peculiar functional phenotype as a reaction to specific signals. Depending on the stimulus that macrophages receive they can acquire an M1 or an M2 phenotype (Murray et al., 2014); however, how autophagy affects these events, especially M1 polarization, is still under debate, because of discrepancy in published data that can be explained by differences in the backgrounds of the macrophages studied and in the experimental settings. The first study addressing directly this issue demonstrated that molecular inhibition of autophagy, by targeting Atg5 gene in C57Bl/6 mice, boosts M1 polarization *in vitro* by significantly increasing M1 markers and proinflammatory cytokine release including TNF-alpha, CCL5, IL6, CCL2, and IL1beta (Liu et al., 2015). These data were confirmed by a following paper showing a lower activation of autophagy in M1 macrophages when compared with M2 and demonstrating that the pharmacological inhibitor of autophagy CQ repolarizes M2 macrophages to an M1 phenotype (Guo et al., 2019). Of notice, once acquired an M1 phenotype, macrophages reduce autophagy and increase glycolysis (Matta and Kumar, 2015). In this context, the role of AKT/mTOR pathway seems to be significant in both autophagy and metabolism. Indeed, in M1 polarized macrophages AKT activation mediates the switch to glycolytic metabolism that leads to suppression of autophagy (Matta and Kumar, 2015). However, in 2017 Esteban-Martinez and collaborators by using a different mouse model (i.e., peritoneal elicited macrophages in CD1 mice) stated that mitophagy contributes to macrophage polarization toward the proinflammatory and more glycolytic M1 phenotype by eliminating mitochondria, but not to M2 macrophage polarization that relies mainly on oxidative phosphorylation (Esteban-Martinez et al., 2017). On the other hand, it seems clear that induction of autophagy supports M2 polarization involving AKT/PI3K pathway, STAT6 and Atg7 (Kapoor et al., 2015; Vergadi et al., 2017; Sanjurjo et al., 2018; Wen et al., 2019; Bo et al., 2020).

Recently, it has been demonstrated that autophagy plays a key role in cytokine production by macrophages. The lack of autophagy, obtained by the genetic depletion of Atg16L1, beclin-1, and LC3, indeed enhances the secretion of interleukin 1beta (IL-1beta) and IL-18 in response to lipopolysaccharide (LPS) and other pathogen-associated molecular patterns (Saitoh et al., 2008; Nakahira et al., 2011). In macrophages, the secretion of IL-1beta and IL-18 is controlled by the signaling platform known as the inflammasome via the release of mitochondrial DNA (mtDNA) and ROS production and the ensuing cleavage and activation of caspase-1. Autophagy by controlling the elimination of dysfunctional mitochondria and the translocation of mtDNA into the cytosol inhibits mitochondrial ROS generation and exerts an anti-inflammatory effect (Nakahira et al., 2011).

### Autophagy and Dendritic Cells

Little is known about the role of autophagy in the generation of DCs. While it does not seem to have a role in the development of immature DCs, however, it seems to contribute to the activation and function of mature DCs, thus indicating autophagy as a key player in the physiological and pathological processes that rely on DCs. Targeting of Atg16L1, a gene associated with inflammatory bowel disease has been demonstrated to boost the immunostimulatory capability of murine mature DCs by increasing the expression of co-stimulatory molecules on the cell surface (Hubbard-Lucey et al., 2014). Autophagy dependent degradation of intracellular materials, including phagocytosed antigens, has been described as a key route for endogenous and exogenous antigens to reach the MHC-II presentation machinery in DCs and activate CD4+ T cells (Dengjel et al., 2005; Munz, 2009; Lee et al., 2010; Keller et al., 2017). Thus autophagy, by regulating MHC-II presentation in DCs, might shape the self-tolerance of CD4+ T cells and trigger CD4+ T cell responses against pathogens and tumors. Moreover, it is clear that autophagy in DCs is required for Treg homeostasis and function both in normal and pathological conditions, as for instance in the development of autoimmune diseases (Niven et al., 2019). In this context, it has to be noted that Treg cells themselves may suppress CD4+ T cell-dependent autoimmune responses through inhibition of autophagy in DCs in a cytotoxic T-lymphocyte-associated protein 4-dependent (CTLA4-dependent) manner (Alissafi et al., 2017).

### Autophagy and T Cells

Lymphocytes (T and B cells) are the essential mediators of the adaptive immune system. All lymphocytes originate from a common progenitor [common lymphoid progenitor (CLP)], derived from the hematopoietic stem cell. In naïve T cells, macroautophagy is active and constitutes a key mechanism for preserving cells homeostasis and survival and adapting cells to intracellular or extracellular environment modifications (Li et al., 2006; Pua et al., 2007). This has been addressed in autophagy gene-deficient T cells, such as cell lacking Atg5, Atg7, beclin-1, or VPS34 (Li et al., 2006; Pua et al., 2007, 2009; Kovacs et al., 2012; Willinger and Flavell, 2012). Indeed, mice with autophagy-deficient T cells displayed a strong decrease in both mature CD4+ and CD8+ cells and the remaining T cells failed to proliferate upon T cell receptor (TCR) stimulation (Pua et al., 2009). In T cells, autophagy acts as a pro-survival process also by eliminating the excess of organelles such as mitochondria and ER. Defective mitophagy in T cells contributes to increase of mitochondrial mass and the ensuing ROS production, therefore prompting the cells to cell death via apoptosis (Pua et al., 2009; Jia and He, 2011). Moreover, the genetic deletion of Atg3, Atg5, and Atg7 results in expanded ER compartments containing increased calcium stores that cannot be depleted properly, thus causing defective calcium influx inside the cells (Jia and He, 2011; Jia et al., 2011). Several studies provided evidence that TCR engagement in CD4+ and CD8+ T cells triggers autophagy activation to support energetic demands for proliferation and cytokines production (Li et al., 2006; Arnold et al., 2014; Botbol et al., 2015; Bantug et al., 2018). Pharmacological or genetic (Atg3, Atg5, or Atg7) inhibition of autophagy has been reported to affect proliferative responses in both CD4+ and CD8+ T cells (Pua et al., 2007; Hubbard et al., 2010). The molecular mechanisms that may induce autophagy in activated T cells are still not completely understood, but multiple pathways seem to be involved. Li et al. (2006) demonstrated that autophagy in TCR activated T cells is affected by 3-MA, an inhibitor of PI3K, as well as JNK inhibitors, and can be enhanced by rapamycin (inhibitor of the mTOR pathway) and zVAD (inhibitor of caspase activity), suggesting a role for all these pathways. More recently, Botbol et al. (2015) reported that intracellular signaling of cytokines such as IL2, IL4, IL7, and IL15 can induce autophagy in CD4+ T cells and demonstrated the involvement of Janus kinase 3 (JAK3). Autophagy has been shown to control also Treg stability and function (Wei et al., 2016). Treg cells have higher autophagy activity than naive CD4+ T cells and Treg cells- specific deletion of Atg7 or Atg5, results in increased cell apoptosis and therefore in the development of autoimmune and inflammatory disorders or tumor resistance (Zhang et al., 2019).

### Autophagy and B Cells

Autophagy has recently emerged as crucial for the maintenance of memory B cells and plasma cells, while its role in the development and survival of naïve B cells needs further elucidation. By using two new mouse models of conditional Atg5 deletion, one under the control of a promoter active early during B-cell development and the other active in mature B cells, Arnold and collaborators drawn the conclusion that basal levels of autophagy are necessary to maintain a normal number of peripheral B cells and central during development of B-1a B cells, tissue-resident, innate-like B cells (Arnold et al., 2016). Subsequently, it has been also demonstrated that B1a B cells have active glycolysis and fatty acid synthesis and depend on autophagy to survive and self-renew (Clarke et al., 2018). Following appropriate stimulation, peripheral B cells in lymphoid follicles may differentiate in the germinal center (GC) B cells, which generate either plasma cells or memory B cells. It has been recently reported that GC B cells are the most active B cells in processing autophagy, which appears to be non-canonical autophagy independent of Atg genes (Martinez-Martin et al., 2017).

Both plasma cells and memory B cells display high levels of autophagy. The elevated secretory activity of plasma cells makes them particularly susceptible to ER stress thus determining the activation of the unfolded protein response (UPR), whose principal aim is to restore ER homeostasis (Li et al., 2019). In response to the challenge of misfolded proteins, autophagy may function as an adaptive ‘self-eating’ process by which excessive intracellular components are encapsulated within autophagosomes and degraded. Pengo et al. (2013), in autophagy-deficient (Atg5f/fCD19-Cre) plasma cells, discovered that autophagy is required for plasma cells maintenance and humoral immunity, by limiting ER expansion and immunoglobulin synthesis while sustaining energy metabolism and viability. In memory B cells autophagy deficiency leads to a significant decline of cell number accompanied by accumulation of abnormal mitochondria, excessive ROS production and oxidative damage and Ab-dependent immunological memory (Chen M. et al., 2014; Chen et al., 2015). Of notice, autophagy is dispensable for the initial formation of memory B cells, but it is necessary for their long-term maintenance, via the upregulation of the expression of BCL2 and autophagy genes, starting from the transcription factors FoxO1 and FoxO3 (Chen et al., 2015). Finally, autophagy has been demonstrated to be central in MHC-II-dependent antigen presentation by B cells to CD4+ T cells. In their role of APCs B cells can induce T cell tolerance and, in case of presenting self-antigens, they can be involved in the development of autoimmune diseases. Moreover, priming of helper T cells by B cells provides instructive signals for B cells terminal differentiation into memory or plasma cells secreting high-affinity antibodies (Rodriguez-Pinto, 2005). In B cells as APCs, autophagy is indeed implicated in the processing of viral antigens for MHC-II-mediated presentation (Paludan et al., 2005), and optimizes B cell receptor signaling in response to nucleic acid antigens (Chaturvedi et al., 2008). Recently, it has been reported that Atg5 is involved in B cell receptor trafficking and in the recruitment of lysosomes and MHC-II compartment in B cells, thus suggesting that autophagy may control the B cell activation steps required for the humoral immune response against particulate antigens (Arbogast et al., 2019).

## Autophagy and Brain Homeostasis

It is well known that the development, survival and function of nervous system strongly depend on autophagy, which plays a crucial role to maintain neuronal homeostasis and functioning (Yamamoto and Yue, 2014; Ariosa and Klionsky, 2016). In fact, as post-mitotic cells, neurons cannot remove dysfunctional cellular components by cell division. Conversely, they require specific processes aimed to preserve their viability during lifetime. To better understand the importance of autophagy for neurons, we have to consider that differently from frequently replaced cells, most of the neurons are generated during embryogenesis and have to maintain their functionality for the entire lifespan of the individual. The critical relationship between neurons and autophagy is demonstrated by the detrimental impact of the deletion from the neural lineage of specific Atg genes, which results in cytoplasmic inclusions and increased risk of neurodegeneration, even without concomitant pathological processes. Moreover, growing evidence suggests the involvement of autophagy in several pathologies characterized by neuronal dysfunction without cell death. In line with these observations, a better knowledge of the mechanisms underlying autophagy in neurons would be crucial to develop potential therapeutic strategies able to preserve and/or protect neuronal health. Although in this review we aim to specifically describe the involvement of autophagy in neuronal health and function, we have to be aware that other cells of the nervous systems such as glial populations, i.e., astrocytes, oligodendrocytes, Schwann cells and microglia cells are under the control of this process [for review (Kulkarni et al., 2018)]. The comprehension of the mechanisms by which autophagy occurs within the nervous system, both in neurons and in non-neuronal cells, may provide crucial information to address autophagy as potential therapeutic target. Table 4 summarizes the present understanding on the role of autophagy in brain homeostasis, focusing on the genetic or pharmacological approaches used to modulate the autophagic process both *in vitro* and *in vivo*. All the data presented in Table 4 are deeply discussed in the sections below.

**TABLE 4:** A summary of the current knowledge on the role of autophagy in the maintenance of brain behavior and functions.

*In vitro* genetic or pharmacological modulation	Autophagy	Cell type	Biological effect	References
FIP200–/– mouse embryonic fibroblasts	↓	Embryonic fibroblasts	Reduced autophagosome formation, cell degeneration	Hara et al., 2008
Lymphoblastoid cell lines of ataxia patients	↓	Lymphoblastoid cell lines	ATG5 mutation	Kim et al., 2016
ATG5 silencing	↓	Cortical neural progenitor cells	Inhibited differentiation	Lv et al., 2014
ATG7 or ATG16L1 silencing	↓	HEK cells	Modulation of Notch signaling and stem cell differentiation	Wu et al., 2016
***In vivo* genetic or**				
***pharmacological modulation***				
Brain ATG5–/– mice	↓	Several brain regions	Cerebral cortex atrophy, neuronal loss, motor and behavioral deficits	Komatsu et al., 2006
Neural-cell-specific ATG5–/– mice	↓	Several brain regions	Cell degeneration, protein aggregation, neurological phenotype	Hara et al., 2006
Neural-specific conditional FIP200–/– mice	↓	Cerebellum	Reduced autophagosome formation, cell degeneration, apoptosis, neuronal loss, protein aggregation, mitochondrial impairment, cerebellar ataxia, animal lethality	Liang et al., 2010
Purkinje cell-specific conditional ATG5–/– mice	↓	Purkinje cell	Axonal swelling, autophagosome-like vesicles accumulation, cell degeneration, ataxia	Nishiyama et al., 2007
Purkinje cell-specific conditional ATG7–/– mice	↓	Cerebellum, Purkinje cell	Axonal dystrophy, cell death, locomotion and motor coordination impairment	Komatsu et al., 2007
ATG3*–/–* mice, *A*TG12*–/–* mice, ATG16L1 mutant mice	↓	–	Animal lethality	Saitoh et al., 2008; Sou et al., 2008; Malhotra et al., 2015
ATG5–/– mice with re-expression of ATG5 in the brain	–	Brain	Rescue of ATG5–/– mice	Yoshii et al., 2016
Eva1a–/– mice	↓	Brain	Impaired neurogenesis	Wu et al., 2016
WDFY3 mutant mice	↓	Mitochondria	Mitophagy, impaired mitochondrial function	Napoli et al., 2018
Brain of ASds patients	↓	Temporal lobe, layer V pyramidal neurons	Increased dendritic spine density, reduced developmental spine pruning	Tang et al., 2014
Conditional neuronal ATG7–/– mice	↓	Pyramidal neurons	Spine pruning defect, increased dendritic spines	Tang et al., 2014
Dopamine neuron-specific ATG7–/– mice	↓	Corticostriatal slices, dopaminergic neurons	Abnormally large dopaminergic axonal profiles, increased neurotransmitter release, rapid presynaptic recovery	Hernandez et al., 2012
Microglia-specific ATG7–/– mice	↓	Brain	ASDs-like phenotype, increased dendritic spines and synaptic markers, altered connectivity between brain regions	Kim et al., 2017
Systemic administration of young plasma into aged mice	↑	Hippocampal neurons	Recovery memory deficits	Glatigny et al., 2019
Drosophila brain	↓	Drosophila learning center (mushroom body)	Memory impairment, increased metaplasticity	Bhukel et al., 2019
Amitriptyline treated mice	↓	Hippocampal lysosomes	Sphingomyelin accumulation, ceramide increase	Gulbins et al., 2018

### Autophagy and Neuronal Survival

The role of autophagy in neuronal survival has been demonstrated by using mice and/or other models with whole-body or conditional/selective knockouts of Atg genes. For example, deletion of genes codifying proteins involved in the formation of autophagosome within the nervous system such as Atg5 and Atg7 caused axon swelling and neuron death (Hara et al., 2006; Komatsu et al., 2006). Specifically, neuronal loss was detected in the Purkinje cell layer of the cerebellum as well as in the pyramidal cells of cerebral cortex and hippocampus (Hara et al., 2006; Komatsu et al., 2006). Interestingly, inhibition of autophagy was paralleled by increased ubiquitin-positive aggregates, an effect restricted to neurons with no impact on the surrounding glia (Hara et al., 2006; Komatsu et al., 2006). It is important to note that the loss of autophagy differently affects the various brain regions. For example, despite their susceptibility to the consequences of compromised autophagy, Purkinje cells were not characterized by aggregates (Hara et al., 2006). Degeneration and death of Purkinje cells have been also observed in response to neural-specific deletion of FIP200, a protein required for the initiation of autophagosome formation (Hara et al., 2008). The negative impact of autophagy loss was associated with behavioral alterations such as cerebellar ataxia, motor deficits and even animal lethality (Hara et al., 2006; Komatsu et al., 2006; Liang et al., 2010). Since Atg5 or Atg7 deletion affects both neuron and glial cell precursors, neuron-specific knockout mice were developed. Specifically, Atg5 or Atg7 genetic inactivation within Purkinje cells caused gradual axon degeneration and neuronal death paralleled by deficit in motor coordination (Komatsu et al., 2007; Nishiyama et al., 2007). One of the most frequent behavioral consequences of neuronal autophagy deficiency in mice is neonatal lethality, an effect observed not only in response to Atg5 deletion, but also in Atg3, Atg7, Atg12, and Atg16L1 null mice (Komatsu et al., 2005; Saitoh et al., 2008; Sou et al., 2008; Malhotra et al., 2015). Recently, it has been demonstrated that an almost complete rescue from neonatal lethality was possible by re-expressing Atg5 only in neuron, suggestive of neuronal dysfunction as the primary cause of neonatal lethality (Yoshii et al., 2016).

As previously mentioned, in human autophagy deficiency cell death has been associated with neurodegenerative disorders. For example, childhood ataxia, a movement disorder linked with degeneration of cerebellar Purkinje cells has been recently demonstrated to be caused by a mutation in Atg5 that is able to reduce autophagic flux (Kim et al., 2016). Similarly, mutations in autophagy receptors and/or regulatory proteins have been linked to several neurodegenerative diseases (Frake et al., 2015). Most of these disorders such as Amyotrophic Lateral Sclerosis, Alzheimer’s, Parkinson’s, and Huntington’s Diseases have been characterized by neuronal autophagosomes accumulation (Nixon et al., 2005; Son et al., 2012; Menzies et al., 2017). This effect might be due to an unbalance between autophagosome formation and clearance. Although the specific mechanism is currently unknown, the latter hypothesis seems more feasible. Indeed, neurodegenerative experimental models showed compromised axonal transport and defective lysosomal function, alterations that may affect the clearance ability leading to neuronal death (Millecamps and Julien, 2013; Maday and Holzbaur, 2014; Gowrishankar et al., 2015).

### Autophagy and Neurogenesis

In the last years, there has been a growing interest in the role of autophagy in the maintenance of neuronal stem cells as well as in the proliferation of neural progenitors. In particular, it has been reported that the developing central nervous system (CNS) expresses core autophagy genes (Wu et al., 2013) and that neurogenesis in the developing embryo is regulated by autophagy (Kuma et al., 2017). Specifically, neural progenitor cells (NPCs) in the embryonic mouse cortex express Atg genes, whose silencing results in reduced neuronal proliferation, altered growth and branching of cortical neurons, increased cells in the subventricular (SVZ) and ventricular zones (Lv et al., 2014). A similar effect was found in another study using Atg16L1 mice, in which reduced cortical plate and enlarged SVZ were observed (Wu et al., 2016). Several mediators are thought potential players of these effects. Specifically, it has been suggested the involvement of the lysosome and ER- associated protein EVA1 (transmembrane protein 166, TMEM166 protein) largely distributed in the brain during neurogenesis and involved in autophagy and apoptosis (Li et al., 2016). Loss of Nestin-expressing neuronal stem cells (NSCs) and decreased self-renewal and differentiation were observed in the cortex of EVA1 conditional knockout mice, effect due to the activation of mTOR pathway (Li et al., 2016).

The role of autophagy in neurogenesis is supported by the autosomal dominant human microcephaly observed in response to mutations of specific autophagy genes (Kadir et al., 2016). Specifically, the mutations are identified on a scaffolding protein responsible for the degradation of ubiquitinated aggregate-prone proteins by autophagy (Filimonenko et al., 2010) and clearance of mitochondria via mitophagy (Napoli et al., 2018). Interestingly, studies in Drosophila, reported that the expression of the mutant protein is paralleled by a marked reduction of the brain volume (Kadir et al., 2016).

Moreover, mild non-specific neurodevelopmental delay has been associated with further mutations that result in truncated proteins or missense heterozygous mutations and may lead to microcephaly or macrocephaly, autism spectrum disorder, and attention deficit hyperactivity disorder depending on the protein domain in which the mutation occurs (Le Duc et al., 2019).

Interestingly, the main clinical abnormalities associated with genetic mutations in autophagy genes are developmental delay, cognitive decline, and functional deficits while only minor structural alterations were observed [for detailed examination, (Fleming and Rubinsztein, 2020)]. Nevertheless, further studies are demanded to clarify the mechanisms underlying the impact of these mutations.

### Autophagy and Neuronal Plasticity

Despite the contribution of autophagy in the regulation of developing nervous system, this process is critical also for the mature nervous system. In particular, autophagy exerts an important role in the maintenance of the so-called neuronal plasticity: the ability of the neuronal cells to perceive, respond and adapt to any internal or external, beneficial, or detrimental stimulus. It is well-known that a variety of functions require this capability: learning and memory, cognition, several intellectual abilities, adaptive behaviors, injury repair. All these behaviors involve structural remodeling of the neuron itself and the circuits in which is involved trough axonal growth, synaptic assembly, dendritic spine formation, and pruning (Lieberman and Sulzer, 2020).

Again, major findings on the role of autophagy in neuronal plasticity derive from studies with mutant animals for core autophagy genes. For example, conditional knockout mice with neuronal Atg7 deletion display pyramidal neurons with increased dendritic spines due to spine pruning defect (Tang et al., 2014). Moreover, Atg7 deletion in mouse dopaminergic neurons induces not only structural alteration such as larger axonal profiles but also enhanced stimulus-evoked dopamine release and rapid presynaptic recovery, suggesting a potential regulation of synaptic vesicle turnover (Hernandez et al., 2012). As previously mentioned, given the mutual regulation between neurons and glia, glial cells are involved in the synaptic effect of autophagy.

Specifically, conditional Atg7-knockout mice shown impaired synaptosome degradation, increased dendritic spines and synaptic markers, and altered connectivity paralleled by loss of microglial autophagy (Kim et al., 2017). The mutual influence between autophagy and synaptic machinery has been demonstrated by studies indicating that gain of function or loss of function of the synaptic protein Bassoon may suppress or enhance autophagy through direct interaction with Atg5 (Okerlund et al., 2017; Vanhauwaert et al., 2017). Moreover, blockade of autophagy at the presynaptic terminal may be obtained by deletion of the synaptic protein synaptojanin (Okerlund et al., 2017; Vanhauwaert et al., 2017).

In support of the involvement of autophagy in memory, it has been reported that it is possible to partially recover memory deficit in aged animals by inducing autophagy. In particular, the administration to old animals of plasma obtained from young animals improved memory decline with the involvement of bone-derived osteocalcin, a hormonal regulator of hippocampal memory (Glatigny et al., 2019). A similar effect was observed in Drosophila, where autophagy protects from the memory impairment associated with the expansion of the presynaptic active zone (Bhukel et al., 2019). Despite these observations, exaggerate increased autophagy may be deleterious, as suggested by the loss of pre- and post-synaptic markers – index of proper synaptic function – in response to the hyperactivation of the positive autophagy regulator, AMPK (Domise et al., 2019). Further studies could be important to clarify this issue, however, we have to keep in mind that autophagy proteins may have a non-canonical function leading to microtubule instability that may affect synaptic structural plasticity (Negrete-Hurtado et al., 2020).

Given the role of compromised neuronal plasticity in the etiopathology of several psychiatric disorders, deficit in autophagy has been postulated to contribute to these diseases. In this context, it has been evaluated the ability of antidepressant drugs to modulate autophagy in different preclinical settings. For example, it has been reported that the Selective Serotonin Reuptake Inhibitor (SSRI) fluoxetine, as well as the TriCyclic Antidepressant (TCA) amitriptyline, stimulate autophagy through a mechanism involving the accumulation of sphingomyelin in lysosomes and Golgi membranes and ceramide in the ER (Gulbins et al., 2018). On the other hand, drugs able to induce autophagy display in mice antidepressant properties, suggesting that the ability to modulate the mood action might also depend on autophagy (Kara et al., 2013). Other drugs, able to reduce IP3 such as the mood stabilizers valproate, lithium, and carbamazepine, induce autophagy via the same mechanism (Sarkar et al., 2005).

As previously mentioned, this evidence clearly supports the potential of autophagy as pharmacological target for several diseases of the nervous systems, not only neurodegenerative but also psychiatric.

## Conclusions

Autophagy is often considered as a cell death mechanism in the mammalian system, however, in the last decade, the thorough characterization of macroautophagy at molecular level and the development of reliable methods to monitor and manipulate autophagic activity both *in vitro* and *in vivo* have led to outstanding results in understanding the role of autophagy on tissue pathophysiology.

**FIGURE 1:**
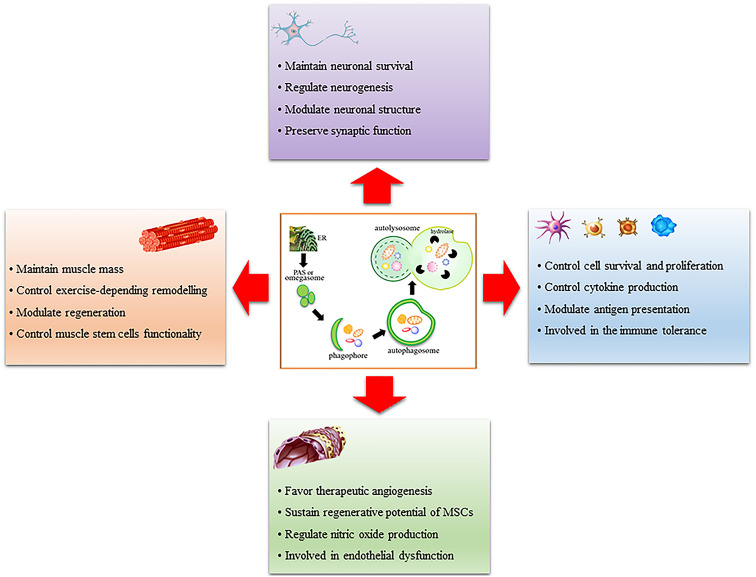
Schematic representation of the role of autophagy in muscle, immune system, endothelium, and brain. Autophagy is a multi-step process that involves phagophore formation from the endoplasmic reticulum-associated structure called omegasome or phagophore assembly site (PAS). This structure grows around the material to be eliminated and forms a characteristic double-membrane vesicle called autophagosome. Mature autophagosome fuses with lysosomes to form autolysosome wherein the material is digested by lysosomal hydrolases. Autophagy regulates many functions in each tissue analyzed, it is important for their remodeling after stimuli or stress conditions and it is altered in many pathologic conditions.

As reported in this review, it is now clear that autophagy is an essential process for the homeostasis of several tissues driving their development, differentiation and ability to remodel after stimuli or under stress conditions. Of note, autophagy alterations are associated with many diseases in each tissue analyzed uncovering the cardinal importance of both basal and inducible autophagy for the maintenance of tissue homeostasis. With this review, we provide a suitable framework of the importance of autophagy in endothelium, muscle, immune systems and brain, as schematically reported in Figure 1. These are very different tissues with a diverse organization and activity and it is worth mentioning that, in each of these, autophagy is relevant and crucial; thereby this should drive scientists to continue studying autophagy in still unexplored fields or deepen some preliminary observations to fully understand autophagy’s role.

## Author Contributions

CDP conceptualized the review and wrote the introduction, conclusions, and Section “Autophagy and Skeletal Muscle Homeostasis.” CP wrote and revised Section “Autophagy and Immune System Homeostasis.” MGC wrote and revised Section “Autophagy and Endothelium Homeostasis.” RM wrote and revised Section “Autophagy and Brain Homeostasis.” All authors read and approved the final manuscript.

## Conflict of Interest

The authors declare that the research was conducted in the absence of any commercial or financial relationships that could be construed as a potential conflict of interest.

## References

[B1] Aas S. N. Tommerbakke D. Godager S. Nordseth M. Armani A. Sandri M. (2020). Effects of acute and chronic strength training on skeletal muscle autophagy in frail elderly men and women. *Exp. Gerontol.* 142:111122 10.1016/j.exger.2020.111122 33132146

[B2] Agnello M. Bosco L. Chiarelli R. Martino C. Roccheri M. C. (2015). “The role of autophagy and apoptosis during embryo development,” in *Cell Death - Autophagy, Apoptosis and Necrosis*, ed. Ntuli T. M. (Reijeka: InTech), 83–112. 10.5772/61765

[B3] Alissafi T. Banos A. Boon L. Sparwasser T. Ghigo A. Wing K. (2017). Tregs restrain dendritic cell autophagy to ameliorate autoimmunity. *J. Clin. Invest.* 127 2789–2804. 10.1172/JCI92079 28581446 PMC5490766

[B4] An Y. Liu W. J. Xue P. Ma Y. Zhang L. Q. Zhu B. (2018). Autophagy promotes MSC-mediated vascularization in cutaneous wound healing via regulation of VEGF secretion. *Cell Death Dis.* 9:58. 10.1038/s41419-017-0082-8 29352190 PMC5833357

[B5] Andersen M. H. Schrama D. Thor Straten P. Becker J. C. (2006). Cytotoxic T cells. *J. Invest. Dermatol.* 126 32–41. 10.1038/sj.jid.5700001 16417215

[B6] Arbogast F. Arnold J. Hammann P. Kuhn L. Chicher J. Murera D. (2019). ATG5 is required for B cell polarization and presentation of particulate antigens. *Autophagy* 15 280–294. 10.1080/15548627.2018.1516327 30196744 PMC6333460

[B7] Ariosa A. R. Klionsky D. J. (2016). Autophagy core machinery: overcoming spatial barriers in neurons. *J. Mol. Med.* 94 1217–1227. 10.1007/s00109-016-1461-9 27544281 PMC5071157

[B8] Arnold C. R. Pritz T. Brunner S. Knabb C. Salvenmoser W. Holzwarth B. (2014). T cell receptor-mediated activation is a potent inducer of macroautophagy in human CD8(+)CD28(+) T cells but not in CD8(+)CD28(−) T cells. *Exp. Gerontol.* 54 75–83. 10.1016/j.exger.2014.01.018 24468331

[B9] Arnold J. Murera D. Arbogast F. Fauny J. D. Muller S. Gros F. (2016). Autophagy is dispensable for B-cell development but essential for humoral autoimmune responses. *Cell Death Differ.* 23 853–864. 10.1038/cdd.2015.149 26586568 PMC4832104

[B10] Axe E. L. Walker S. A. Manifava M. Chandra P. Roderick H. L. Habermann A. (2008). Autophagosome formation from membrane compartments enriched in phosphatidylinositol 3-phosphate and dynamically connected to the endoplasmic reticulum. *J. Cell Biol.* 182 685–701. 10.1083/jcb.200803137 18725538 PMC2518708

[B11] Bantug G. R. Galluzzi L. Kroemer G. Hess C. (2018). The spectrum of T cell metabolism in health and disease. *Nat. Rev. Immunol.* 18 19–34. 10.1038/nri.2017.99 28944771

[B12] Bargiela A. Cerro-Herreros E. Fernandez-Costa J. M. Vilchez J. J. Llamusi B. Artero R. (2015). Increased autophagy and apoptosis contribute to muscle atrophy in a myotonic dystrophy type 1 *Drosophila* model. *Dis. Model Mech.* 8 679–690. 10.1242/dmm.018127 26092529 PMC4486854

[B13] Bharath L. P. Mueller R. Li Y. Y. Ruan T. Kunz D. Goodrich R. (2014). Impairment of autophagy in endothelial cells prevents shear-stress-induced increases in nitric oxide bioavailability. *Can. J. Physiol. Pharmacol.* 92 605–612. 10.1139/cjpp-2014-0017 24941409 PMC8370712

[B14] Bhukel A. Beuschel C. B. Maglione M. Lehmann M. Juhasz G. Madeo F. (2019). Autophagy within the mushroom body protects from synapse aging in a non-cell autonomous manner. *Nat. Commun.* 10:1318. 10.1038/s41467-019-09262-2 30899013 PMC6428838

[B15] Bloemberg D. Quadrilatero J. (2020). Autophagy displays divergent roles during intermittent amino acid starvation and toxic stress-induced senescence in cultured skeletal muscle cells. *J. Cell Physiol.* 10.1002/jcp.30079 [Epub ahead of print]. 33022071

[B16] Bo Q. Shen M. Xiao M. Liang J. Zhai Y. Zhu H. (2020). 3-Methyladenine alleviates experimental subretinal fibrosis by inhibiting macrophages and M2 polarization through the PI3K/Akt Pathway. *J. Ocul. Pharmacol. Ther*. 36 618–628. 10.1089/jop.2019.0112 32552228

[B17] Botbol Y. Patel B. Macian F. (2015). Common gamma-chain cytokine signaling is required for macroautophagy induction during CD4+ T-cell activation. *Autophagy* 11 1864–1877. 10.1080/15548627.2015.1089374 26391567 PMC4824584

[B18] Boya P. Codogno P. Rodriguez-Muela N. (2018). Autophagy in stem cells: repair, remodelling and metabolic reprogramming. *Development* 145:dev146506. 10.1242/dev.146506 29483129

[B19] Breier G. Breviario F. Caveda L. Berthier R. Schnurch H. Gotsch U. (1996). Molecular cloning and expression of murine vascular endothelial cadherin in early stage development of cardiovascular system. *Blood* 87 630–641. 10.1182/blood.v87.2.630.bloodjournal872630 8555485

[B20] Bronckaers A. Hilkens P. Martens W. Gervois P. Ratajczak J. Struys T. (2014). Mesenchymal stem/stromal cells as a pharmacological and therapeutic approach to accelerate angiogenesis. *Pharmacol. Ther.* 143 181–196. 10.1016/j.pharmthera.2014.02.013 24594234

[B21] Bujak A. L. Crane J. D. Lally J. S. Ford R. J. Kang S. J. Rebalka I. A. (2015). AMPK activation of muscle autophagy prevents fasting-induced hypoglycemia and myopathy during aging. *Cell Metab.* 21 883–890. 10.1016/j.cmet.2015.05.016 26039451 PMC5233441

[B22] Cadore E. L. Casas-Herrero A. Zambom-Ferraresi F. Idoate F. Millor N. Gomez M. (2014). Multicomponent exercises including muscle power training enhance muscle mass, power output, and functional outcomes in institutionalized frail nonagenarians. *Age* 36 773–785. 10.1007/s11357-013-9586-z 24030238 PMC4039263

[B23] Call J. A. Wilson R. J. Laker R. C. Zhang M. Kundu M. Yan Z. (2017). Ulk1-mediated autophagy plays an essential role in mitochondrial remodeling and functional regeneration of skeletal muscle. *Am. J. Physiol. Cell Physiol.* 312 C724–C732. 10.1152/ajpcell.00348.2016 28356270 PMC5494591

[B24] Capasso S. Alessio N. Squillaro T. Di Bernardo G. Melone M. A. Cipollaro M. (2015). Changes in autophagy, proteasome activity and metabolism to determine a specific signature for acute and chronic senescent mesenchymal stromal cells. *Oncotarget* 6 39457–39468. 10.18632/oncotarget.6277 26540573 PMC4741838

[B25] Carmignac V. Svensson M. Korner Z. Elowsson L. Matsumura C. Gawlik K. I. (2011). Autophagy is increased in laminin alpha2 chain-deficient muscle and its inhibition improves muscle morphology in a mouse model of MDC1A. *Hum. Mol. Genet.* 20 4891–4902. 10.1093/hmg/ddr427 21920942

[B26] Castagnaro S. Pellegrini C. Pellegrini M. Chrisam M. Sabatelli P. Toni S. (2016). Autophagy activation in COL6 myopathic patients by a low-protein-diet pilot trial. *Autophagy* 12 2484–2495. 10.1080/15548627.2016.1231279 27656840 PMC5173266

[B27] Castets P. Lin S. Rion N. Di Fulvio S. Romanino K. Guridi M. (2013). Sustained activation of mTORC1 in skeletal muscle inhibits constitutive and starvation-induced autophagy and causes a severe, late-onset myopathy. *Cell Metab.* 17 731–744. 10.1016/j.cmet.2013.03.015 23602450

[B28] Ceccariglia S. Cargnoni A. Silini A. R. Parolini O. (2020). Autophagy: a potential key contributor to the therapeutic action of mesenchymal stem cells. *Autophagy* 16 28–37. 10.1080/15548627.2019.1630223 31185790 PMC6984485

[B29] Chang N. C. Nguyen M. Bourdon J. Risse P. A. Martin J. Danialou G. (2012). Bcl-2-associated autophagy regulator Naf-1 required for maintenance of skeletal muscle. *Hum, Mol. Genet.* 21 2277–2287. 10.1093/hmg/dds048 22343142

[B30] Chang N. T. C. (2020). Autophagy and stem cells: self-eating for self-renewal. *Front. Cell Dev. Biol.* 8:138. 10.3389/Fcell.2020.00138 32195258 PMC7065261

[B31] Chang T. C. Hsu M. F. Wu K. K. (2015). High glucose induces bone marrow-derived mesenchymal stem cell senescence by upregulating autophagy. *PLoS One* 10:e0126537. 10.1371/journal.pone.0126537 25961745 PMC4427318

[B32] Chaturvedi A. Dorward D. Pierce S. K. (2008). The B cell receptor governs the subcellular location of Toll-like receptor 9 leading to hyperresponses to DNA-containing antigens. *Immunity* 28 799–809. 10.1016/j.immuni.2008.03.019 18513998 PMC2601674

[B33] Chen D. Xie J. Fiskesund R. Dong W. Liang X. Lv J. (2018). Chloroquine modulates antitumor immune response by resetting tumor-associated macrophages toward M1 phenotype. *Nat. Commun*, 9:873. 10.1038/s41467-018-03225-9 29491374 PMC5830447

[B34] Chen X. H. He Y. F. Lu F. (2018). Autophagy in stem cell biology: a perspective on stem cell self-renewal and differentiation. *Stem Cells Int.* 2018:9131397. 10.1155/2018/9131397 29765428 PMC5896318

[B35] Chen F. Chen B. Xiao F. Q. Wu Y. T. Wang R. H. Sun Z. W. (2014). Autophagy protects against senescence and apoptosis via the RAS-mitochondria in high-glucose-induced endothelial cells. *Cell Physiol. Biochem.* 33 1058–1074. 10.1159/000358676 24732710

[B36] Chen M. Hong M. J. Sun H. Wang L. Shi X. Gilbert B. E. (2014). Essential role for autophagy in the maintenance of immunological memory against influenza infection. *Nat. Med.* 20 503–510. 10.1038/nm.3521 24747745 PMC4066663

[B37] Chen M. Kodali S. Jang A. Kuai L. Wang J. (2015). Requirement for autophagy in the long-term persistence but not initial formation of memory B cells. *J. Immunol.* 194 2607–2615. 10.4049/jimmunol.1403001 25672753 PMC4355050

[B38] Chen M. L. Yi L. Jin X. Liang X. Y. Zhou Y. Zhang T. (2013). Resveratrol attenuates vascular endothelial inflammation by inducing autophagy through the cAMP signaling pathway. *Autophagy* 9 2033–2045. 10.4161/auto.26336 24145604

[B39] Clarke A. J. Riffelmacher T. Braas D. Cornall R. J. Simon A. K. (2018). B1a B cells require autophagy for metabolic homeostasis and self-renewal. *J. Exp. Med.* 215 399–413. 10.1084/jem.20170771 29326381 PMC5789411

[B40] Cullup T. Kho A. L. Dionisi-Vici C. Brandmeier B. Smith F. Urry Z. (2013). Recessive mutations in EPG5 cause Vici syndrome, a multisystem disorder with defective autophagy. *Nat. Genet.* 45 83–87. 10.1038/ng.2497 23222957 PMC4012842

[B41] de Castro G. S. Simoes E. Lima J. Ortiz-Silva M. Festuccia W. T. Tokeshi F. (2019). Human cachexia induces changes in mitochondria, autophagy and apoptosis in the skeletal muscle. *Cancers* 11:1264. 10.3390/cancers11091264 31466311 PMC6770124

[B42] De Palma C. Morisi F. Cheli S. Pambianco S. Cappello V. Vezzoli M. (2012). Autophagy as a new therapeutic target in Duchenne muscular dystrophy. *Cell Death Dis.* 3:e418. 10.1038/cddis.2012.159 PMC3542595 23152054

[B43] De Palma C. Perrotta C. Pellegrino P. Clementi E. Cervia D. (2014). Skeletal muscle homeostasis in duchenne muscular dystrophy: modulating autophagy as a promising therapeutic strategy. *Front. Aging Neurosci.* 6:188. 10.3389/fnagi.2014.00188 25104934 PMC4109521

[B44] Dengjel J. Schoor O. Fischer R. Reich M. Kraus M. Muller M. (2005). Autophagy promotes MHC class II presentation of peptides from intracellular source proteins. *Proc. Natl. Acad. Sci. U.S.A.* 102 7922–7927. 10.1073/pnas.0501190102 15894616 PMC1142372

[B45] Di Rienzo M. Antonioli M. Fusco C. Liu Y. Mari M. Orhon I. (2019). Autophagy induction in atrophic muscle cells requires ULK1 activation by TRIM32 through unanchored K63-linked polyubiquitin chains. *Sci. Adv.* 5:eaau8857. 10.1126/sciadv.aau8857 31123703 PMC6527439

[B46] Dobrowolny G. Aucello M. Rizzuto E. Beccafico S. Mammucari C. Boncompagni S. (2008). Skeletal muscle is a primary target of SOD1G93A-mediated toxicity. *Cell Metab.* 8 425–436. 10.1016/j.cmet.2008.09.002 19046573

[B47] Domise M. Sauve F. Didier S. Caillerez R. Begard S. Carrier S. (2019). Neuronal AMP-activated protein kinase hyper-activation induces synaptic loss by an autophagy-mediated process. *Cell Death Dis.* 10:221. 10.1038/s41419-019-1464-x 30833547 PMC6399353

[B48] Du J. H. Teng R. J. Guan T. J. Eis A. Kaul S. Konduri G. G. (2012). Role of autophagy in angiogenesis in aortic endothelial cells. *Am. J. Physiol. Cell Physiol.* 302 C383–C391. 10.1152/ajpcell.00164.2011 22031599 PMC3328843

[B49] Eibel H. Kraus H. Sic H. Kienzler A. K. Rizzi M. (2014). B cell biology: an overview. *Curr. Allergy Asthma Rep.* 14:434. 10.1007/s11882-014-0434-8 24633618

[B50] Esteban-Martinez L. Sierra-Filardi E. McGreal R. S. Salazar-Roa M. Marino G. Seco E. (2017). Programmed mitophagy is essential for the glycolytic switch during cell differentiation. *EMBO J.* 36 1688–1706. 10.15252/embj.201695916 28465321 PMC5470043

[B51] Fafian-Labora J. A. Morente-Lopez M. Arufe M. C. (2019). Effect of aging on behaviour of mesenchymal stem cells. *World J. Stem Cells* 11 337–346. 10.4252/wjsc.v11.i6.337 31293716 PMC6600848

[B52] Fan J. Kou X. Jia S. Yang X. Yang Y. Chen N. (2016). Autophagy as a potential target for sarcopenia. *J. Cell Physiol.* 231 1450–1459. 10.1002/jcp.25260 26580995

[B53] Fetalvero K. M. Yu Y. Goetschkes M. Liang G. Valdez R. A. Gould T. (2013). Defective autophagy and mTORC1 signaling in myotubularin null mice. *Mol. Cell Biol.* 33 98–110. 10.1128/MCB.01075-12 23109424 PMC3536306

[B54] Fetterman J. L. Holbrook M. Flint N. Feng B. Breton-Romero R. Linder E. A. (2016). Restoration of autophagy in endothelial cells from patients with diabetes mellitus improves nitric oxide signaling. *Atherosclerosis* 247 207–217. 10.1016/j.atherosclerosis.2016.01.043 26926601 PMC4913892

[B55] Fiacco E. Castagnetti F. Bianconi V. Madaro L. De Bardi M. Nazio F. (2016). Autophagy regulates satellite cell ability to regenerate normal and dystrophic muscles. *Cell Death Differ.* 23 1839–1849. 10.1038/cdd.2016.70 27447110 PMC5071573

[B56] Filimonenko M. Isakson P. Finley K. D. Anderson M. Jeong H. Melia T. J. (2010). The selective macroautophagic degradation of aggregated proteins requires the PI3P-binding protein Alfy. *Mol. Cell* 38 265–279. 10.1016/j.molcel.2010.04.007 20417604 PMC2867245

[B57] Filippi I. Saltarella I. Aldinucci C. Carraro F. Ria R. Vacca A. (2018). Different adaptive responses to hypoxia in normal and multiple myeloma endothelial cells. *Cell. Physiol. Biochem.* 46 203–212. 10.1159/000488423 29587264

[B58] Fleming A. Rubinsztein D. C. (2020). Autophagy in neuronal development and plasticity. *Trends Neurosci.* 43 767–779. 10.1016/j.tins.2020.07.003 32800535

[B59] Frake R. A. Ricketts T. Menzies F. M. Rubinsztein D. C. (2015). Autophagy and neurodegeneration. *J. Clin. Invest.* 125 65–74. 10.1172/JCI73944 25654552 PMC4382230

[B60] Fukuda T. Roberts A. Ahearn M. Zaal K. Ralston E. Plotz P. H. (2006). Autophagy and lysosomes in Pompe disease. *Autophagy* 2 318–320. 10.4161/auto.2984 16874053

[B61] Fuqua J. D. Mere C. P. Kronemberger A. Blomme J. Bae D. Turner K. D. (2019). ULK2 is essential for degradation of ubiquitinated protein aggregates and homeostasis in skeletal muscle. *FASEB J.* 33 11735–11745. 10.1096/fj.201900766R 31361156 PMC6902739

[B62] Garcia-Prat L. Martinez-Vicente M. Perdiguero E. Ortet L. Rodriguez-Ubreva J. Rebollo E. (2016). Autophagy maintains stemness by preventing senescence. *Nature* 529 37–42. 10.1038/nature16187 26738589

[B63] Gkikas I. Palikaras K. Tavernarakis N. (2018). The Role of mitophagy in innate immunity. *Front. Immunol.* 9:1283. 10.3389/fimmu.2018.01283 29951054 PMC6008576

[B64] Glatigny M. Moriceau S. Rivagorda M. Ramos-Brossier M. Nascimbeni A. C. Lante F. (2019). Autophagy is required for memory formation and reverses age-related memory decline. *Curr Biol.* 29 435.e8–448.e8 10.1016/j.cub.2018.12.021 30661803

[B65] Glimcher L. H. Murphy K. M. (2000). Lineage commitment in the immune system: the T helper lymphocyte grows up. *Genes Dev.* 14 1693–1711. 10898785

[B66] Gowrishankar S. Yuan P. Wu Y. Schrag M. Paradise S. Grutzendler J. (2015). Massive accumulation of luminal protease-deficient axonal lysosomes at Alzheimer’s disease amyloid plaques. *Proc. Natl. Acad. Sci. U.S.A.* 112 E3699–E3708. 26124111 10.1073/pnas.1510329112 PMC4507205

[B67] Grumati P. Coletto L. Sabatelli P. Cescon M. Angelin A. Bertaggia E. (2010). Autophagy is defective in collagen VI muscular dystrophies, and its reactivation rescues myofiber degeneration. *Nat. Med.* 16 1313–1320. 10.1038/nm.2247 21037586

[B68] Grumati P. Coletto L. Schiavinato A. Castagnaro S. Bertaggia E. Sandri M. (2011). Physical exercise stimulates autophagy in normal skeletal muscles but is detrimental for collagen VI-deficient muscles. *Autophagy* 7 1415–1423. 10.4161/auto.7.12.17877 22024752 PMC3288016

[B69] Gulbins A. Schumacher F. Becker K. A. Wilker B. Soddemann M. Boldrin F. (2018). Antidepressants act by inducing autophagy controlled by sphingomyelin-ceramide. *Mol. Psychiatry* 23 2324–2346. 10.1038/s41380-018-0090-9 30038230 PMC6294742

[B70] Guo F. X. Li X. H. Peng J. Tang Y. L. Yang Q. Liu L. S. (2014). Autophagy regulates vascular endothelial cell eNOS and ET-1 expression induced by laminar shear stress in an Ex Vivo perfused system. *Ann. Biomed. Eng.* 42 1978–1988. 10.1007/s10439-014-1033-5 24838486

[B71] Guo Y. Feng Y. Cui X. Wang Q. Pan X. (2019). Autophagy inhibition induces the repolarisation of tumour-associated macrophages and enhances chemosensitivity of laryngeal cancer cells to cisplatin in mice. *Cancer Immunol. Immunother.* 68 1909–1920. 10.1007/s00262-019-02415-8 31641796 PMC11028258

[B72] Hara T. Nakamura K. Matsui M. Yamamoto A. Nakahara Y. Suzuki-Migishima R. (2006). Suppression of basal autophagy in neural cells causes neurodegenerative disease in mice. *Nature* 441 885–889. 10.1038/nature04724 16625204

[B73] Hara T. Takamura A. Kishi C. Iemura S. Natsume T. Guan J. L. (2008). FIP200, a ULK-interacting protein, is required for autophagosome formation in mammalian cells. *J. Cell Biol.* 181 497–510. 10.1083/jcb.200712064 18443221 PMC2364687

[B74] Harris J. (2011). Autophagy and cytokines. *Cytokine* 56 140–144. 10.1016/j.cyto.2011.08.022 21889357

[B75] He C. Bassik M. C. Moresi V. Sun K. Wei Y. Zou Z. (2012). Exercise-induced BCL2-regulated autophagy is required for muscle glucose homeostasis. *Nature* 481 511–515. 10.1038/nature10758 22258505 PMC3518436

[B76] Hentila J. Ahtiainen J. P. Paulsen G. Raastad T. Hakkinen K. Mero A. A. (2018). Autophagy is induced by resistance exercise in young men, but unfolded protein response is induced regardless of age. *Acta Physiol.* 224:e13069. 10.1111/apha.13069 29608242

[B77] Hernandez D. Torres C. A. Setlik W. Cebrian C. Mosharov E. V. Tang G. (2012). Regulation of presynaptic neurotransmission by macroautophagy. *Neuron* 74 277–284. 10.1016/j.neuron.2012.02.020 22542182 PMC3578406

[B78] Ho T. T. Warr M. R. Adelman E. R. Lansinger O. M. Flach J. Verovskaya E. V. (2017). Autophagy maintains the metabolism and function of young and old stem cells. *Nature* 543 205–210. 10.1038/nature21388 28241143 PMC5344718

[B79] Hsieh H. J. Liu C. A. Huang B. Tseng A. H. H. Wang D. L. (2014). Shear-induced endothelial mechanotransduction: the interplay between reactive oxygen species (ROS) and nitric oxide (NO) and the pathophysiological implications. *J. Biomed. Sci.* 21:3. 10.1186/1423-0127-21-3 24410814 PMC3898375

[B80] Hubbard V. M. Valdor R. Patel B. Singh R. Cuervo A. M. Macian F. (2010). Macroautophagy regulates energy metabolism during effector T cell activation. *J. Immunol.* 185 7349–7357. 10.4049/jimmunol.1000576 21059894 PMC3046774

[B81] Hubbard-Lucey V. M. Shono Y. Maurer K. West M. L. Singer N. V. Ziegler C. G. (2014). Autophagy gene Atg16L1 prevents lethal T cell alloreactivity mediated by dendritic cells. *Immunity* 41 579–591. 10.1016/j.immuni.2014.09.011 25308334 PMC4237219

[B82] Hughes W. E. Beyer A. M. Gutterman D. D. (2020). Vascular autophagy in health and disease. *Basic Res. Cardiol.* 115:41. 10.1007/s00395-020-0802-6 32506214 PMC12306491

[B83] Italiani P. Boraschi D. (2014). From Monocytes to M1/M2 macrophages: phenotypical vs. functional differentiation. *Front. Immunol.* 5:514. 10.3389/fimmu.2014.00514 25368618 PMC4201108

[B84] Jacquel A. Obba S. Boyer L. Dufies M. Robert G. Gounon P. (2012). Autophagy is required for CSF-1-induced macrophagic differentiation and acquisition of phagocytic functions. *Blood* 119 4527–4531. 10.1182/blood-2011-11-392167 22452982

[B85] Jakovljevic J. Harrell C. R. Fellabaum C. Arsenijevic A. Jovicic N. Volarevic V. (2018). Modulation of autophagy as new approach in mesenchymal stem cell-based therapy. *Biomed. Pharmacother.* 104 404–410. 10.1016/j.biopha.2018.05.061 29787987

[B86] Jarosz-Biej M. Kaminska N. Matuszczak S. Cichon T. Pamula-Pilat J. Czapla J. (2018). M1-like macrophages change tumor blood vessels and microenvironment in murine melanoma. *PLoS One* 13:e0191012. 10.1371/journal.pone.0191012 29320562 PMC5761928

[B87] Jia W. He Y. W. (2011). Temporal regulation of intracellular organelle homeostasis in T lymphocytes by autophagy. *J. Immunol.* 186 5313–5322. 10.4049/jimmunol.1002404 21421856

[B88] Jia W. Pua H. H. Li Q. J. He Y. W. (2011). Autophagy regulates endoplasmic reticulum homeostasis and calcium mobilization in T lymphocytes. *J. Immunol.* 186 1564–1574. 10.4049/jimmunol.1001822 21191072 PMC3285458

[B89] Jokl E. J. Blanco G. (2016). Disrupted autophagy undermines skeletal muscle adaptation and integrity. *Mamm Genome* 27 525–537. 10.1007/s00335-016-9659-2 27484057 PMC5110612

[B90] Kadir R. Harel T. Markus B. Perez Y. Bakhrat A. Cohen I. (2016). ALFY-Controlled DVL3 autophagy regulates Wnt signaling, determining human brain size. *PLoS Genet.* 12:e1005919 10.1371/journal.pgen PMC4805177 27008544

[B91] Kapoor N. Niu J. Saad Y. Kumar S. Sirakova T. Becerra E. (2015). Transcription factors STAT6 and KLF4 implement macrophage polarization via the dual catalytic powers of MCPIP. *J. Immunol.* 194 6011–6023. 10.4049/jimmunol.1402797 25934862 PMC4458412

[B92] Kara N. Z. Toker L. Agam G. Anderson G. W. Belmaker R. H. Einat H. (2013). Trehalose induced antidepressant-like effects and autophagy enhancement in mice. *Psychopharmacology* 229 367–375. 10.1007/s00213-013-3119-4 23644913

[B93] Keller C. W. Sina C. Kotur M. B. Ramelli G. Mundt S. Quast I. (2017). ATG-dependent phagocytosis in dendritic cells drives myelin-specific CD4(+) T cell pathogenicity during CNS inflammation. *Proc. Natl. Acad. Sci. U.S.A.* 114 E11228–E11237. 10.1073/pnas.1713664114 29233943 PMC5748192

[B94] Kim H. J. Cho M. H. Shim W. H. Kim J. K. Jeon E. Y. Kim D. H. (2017). Deficient autophagy in microglia impairs synaptic pruning and causes social behavioral defects. *Mol. Psychiatry* 22 1576–1584. 10.1038/mp.2016.103 27400854 PMC5658669

[B95] Kim H. S. Montana V. Jang H. J. Parpura V. Kim J. A. (2013). Epigallocatechin Gallate (EGCG) stimulates autophagy in vascular endothelial cells A POTENTIAL ROLE FOR REDUCING LIPID ACCUMULATION. *J. Biol. Chem.* 288 22693–22705. 10.1074/jbc.M113.477505 23754277 PMC3829354

[B96] Kim K. H. Jeong Y. T. Oh H. Kim S. H. Cho J. M. Kim Y. N. (2013). Autophagy deficiency leads to protection from obesity and insulin resistance by inducing Fgf21 as a mitokine. *Nat. Med.* 19 83–92. 10.1038/nm.3014 23202295

[B97] Kim J. Kundu M. Viollet B. Guan K. L. (2011). AMPK and mTOR regulate autophagy through direct phosphorylation of Ulk1. *Nat. Cell Biol.* 13 132–141. 10.1038/ncb2152 21258367 PMC3987946

[B98] Kim M. Sandford E. Gatica D. Qiu Y. Liu X. Zheng Y. (2016). Mutation in ATG5 reduces autophagy and leads to ataxia with developmental delay. *eLife* 5:e12245. 10.7554/eLife.12245 26812546 PMC4786408

[B99] Klionsky D. J. (2007). Autophagy: from phenomenology to molecular understanding in less than a decade. *Nat. Rev. Mol. Cell Biol.* 8 931–937. 10.1038/nrm2245 17712358

[B100] Kofler N. M. Shawber C. J. Kangsamaksin T. Reed H. O. Galatioto J. Kitajewski J. (2011). Notch signaling in developmental and tumor angiogenesis. *Genes Cancer* 2 1106–1116. 10.1177/1947601911423030 22866202 PMC3411124

[B101] Komatsu M. Waguri S. Chiba T. Murata S. Iwata J. Tanida I. (2006). Loss of autophagy in the central nervous system causes neurodegeneration in mice. *Nature* 441 880–884. 10.1038/nature04723 16625205

[B102] Komatsu M. Waguri S. Ueno T. Iwata J. Murata S. Tanida I. (2005). Impairment of starvation-induced and constitutive autophagy in Atg7-deficient mice. *J. Cell Biol.* 169 425–434. 10.1083/jcb.200412022 15866887 PMC2171928

[B103] Komatsu M. Wang Q. J. Holstein G. R. Friedrich V. L. Jr. Iwata J. Kominami E. (2007). Essential role for autophagy protein Atg7 in the maintenance of axonal homeostasis and the prevention of axonal degeneration. *Proc. Natl. Acad. Sci. U.S.A.* 104 14489–14494. 10.1073/pnas.0701311104 17726112 PMC1964831

[B104] Kovacs J. R. Li C. Yang Q. Li G. Garcia I. G. Ju S. (2012). Autophagy promotes T-cell survival through degradation of proteins of the cell death machinery. *Cell Death Differ.* 19 144–152. 10.1038/cdd.2011.78 21660048 PMC3252822

[B105] Kroemer G. Marino G. Levine B. (2010). Autophagy and the integrated stress response. *Mol. Cell* 40 280–293. 10.1016/j.molcel.2010.09.023 20965422 PMC3127250

[B106] Kulkarni A. Chen J. Maday S. (2018). Neuronal autophagy and intercellular regulation of homeostasis in the brain. *Curr. Opin. Neurobiol.* 51 29–36. 10.1016/j.conb.2018.02.008 29529415

[B107] Kuma A. Komatsu M. Mizushima N. (2017). Autophagy-monitoring and autophagy-deficient mice. *Autophagy* 13 1619–1628. 10.1080/15548627.2017.1343770 28820286 PMC5640176

[B108] Le Duc D. Giulivi C. Hiatt S. M. Napoli E. Panoutsopoulos A. Harlan De Crescenzo A. (2019). Pathogenic WDFY3 variants cause neurodevelopmental disorders and opposing effects on brain size. *Brain* 142 2617–2630. 10.1093/brain/awz198 31327001 PMC6736092

[B109] Lee H. K. Mattei L. M. Steinberg B. E. Alberts P. Lee Y. H. Chervonsky A. (2010). In vivo requirement for Atg5 in antigen presentation by dendritic cells. *Immunity* 32 227–239. 10.1016/j.immuni.2009.12.006 20171125 PMC2996467

[B110] Lee S. J. Kim H. P. Jin Y. Choi A. M. K. Ryter S. W. (2011). Beclin 1 deficiency is associated with increased hypoxia-induced angiogenesis. *Autophagy* 7 829–839. 10.4161/auto.7.8.15598 21685724 PMC3149693

[B111] Li A. Song N. J. Riesenberg B. P. Li Z. (2019). The emerging roles of endoplasmic reticulum stress in balancing immunity and tolerance in health and diseases: mechanisms and opportunities. *Fron.t Immunol.* 10:3154. 10.3389/fimmu.2019.03154 32117210 PMC7026265

[B112] Li C. Capan E. Zhao Y. Zhao J. Stolz D. Watkins S. C. (2006). Autophagy is induced in CD4+ T cells and important for the growth factor-withdrawal cell death. *J. Immunol.* 177 5163–5168. 10.4049/jimmunol.177.8.5163 17015701

[B113] Li H. Horke S. Forstermann U. (2013). Oxidative stress in vascular disease and its pharmacological prevention. *Trends Pharmacol. Sci.* 34 313–319. 10.1016/j.tips.2013.03.007 23608227

[B114] Li M. Lu G. Hu J. Shen X. Ju J. Gao Y. (2016). EVA1A/TMEM166 regulates embryonic neurogenesis by autophagy. *Stem Cell Rep.* 6 396–410. 10.1016/j.stemcr.2016.01.011 26905199 PMC4788774

[B115] Liang C. C. Wang C. Peng X. Gan B. Guan J. L. (2010). Neural-specific deletion of FIP200 leads to cerebellar degeneration caused by increased neuronal death and axon degeneration. *J. Biol. Chem.* 285 3499–3509. 10.1074/jbc.M109.072389 19940130 PMC2823459

[B116] Liang P. Jiang B. Li Y. Liu Z. Zhang P. Zhang M. (2018). Autophagy promotes angiogenesis via AMPK/Akt/mTOR signaling during the recovery of heat-denatured endothelial cells. *Cell Death Dis.* 9:1152. 10.1038/s41419-018-1194-5 30455420 PMC6242874

[B117] Lieberman O. J. Sulzer D. (2020). The synaptic autophagy cycle. *J. Mol. Biol.* 432 2589–2604. 10.1016/j.jmb.2019.12.028 31866297 PMC7814399

[B118] Lira V. A. Okutsu M. Zhang M. Greene N. P. Laker R. C. Breen D. S. (2013). Autophagy is required for exercise training-induced skeletal muscle adaptation and improvement of physical performance. *FASEB J.* 27 4184–4193. 10.1096/fj.13-228486 23825228 PMC4046188

[B119] Liu K. Zhao E. Ilyas G. Lalazar G. Lin Y. Haseeb M. (2015). Impaired macrophage autophagy increases the immune response in obese mice by promoting proinflammatory macrophage polarization. *Autophagy* 11 271–284. 10.1080/15548627.2015.1009787 25650776 PMC4502775

[B120] Lo Verso F. Carnio S. Vainshtein A. Sandri M. (2014). Autophagy is not required to sustain exercise and PRKAA1/AMPK activity but is important to prevent mitochondrial damage during physical activity. *Autophagy* 10 1883–1894. 10.4161/auto.32154 25483961 PMC4502666

[B121] Lu Q. L. Yao Y. F. Hu Z. K. Hu C. Q. Song Q. X. Ye J. (2016). Angiogenic factor AGGF1 activates autophagy with an essential role in therapeutic angiogenesis for heart disease. *PLoS Biol.* 14:e1002529. 10.1371/journal.pbio.1002529 27513923 PMC4981375

[B122] Lu W. H. Shi Y. X. Ma Z. L. Wang G. Liu L. X. Chuai M. L. (2016). Proper autophagy is indispensable for angiogenesis during chick embryo development. *Cell Cycle* 15 1742–1754. 10.1080/15384101.2016.1184803 27163719 PMC4957570

[B123] Lv X. Jiang H. Li B. Liang Q. Wang S. Zhao Q. (2014). The crucial role of Atg5 in cortical neurogenesis during early brain development. *Sci. Rep.* 4:6010. 10.1038/srep06010 25109817 PMC4127499

[B124] Lyu Z. S. Cao X. N. Wen Q. Mo X. D. Zhao H. Y. Chen Y. H. (2020). Autophagy in endothelial cells regulates their haematopoiesis-supporting ability. *Ebiomedicine* 53:102677. 10.1016/J.Ebiom.2020.102677 32114389 PMC7047195

[B125] Ma Y. Qi M. An Y. Zhang L. Q. Yang R. Doro D. H. (2018). Autophagy controls mesenchymal stem cell properties and senescence during bone aging. *Aging Cell* 17:e12709. 10.1111/acel.12709 29210174 PMC5770781

[B126] Maacha S. Sidahmed H. Jacob S. Gentilcore G. Calzone R. Grivel J. C. (2020). Paracrine mechanisms of mesenchymal stromal cells in angiogenesis. *Stem Cells Int.* 2020:4356359. 10.1155/2020/4356359 32215017 PMC7085399

[B127] Macian F. (2019). Autophagy in T Cell function and aging. *Front. Cell Dev. Biol.* 7:213. 10.3389/fcell.2019.00213 31632966 PMC6783498

[B128] Maday S. Holzbaur E. L. (2014). Autophagosome biogenesis in primary neurons follows an ordered and spatially regulated pathway. *Dev. Cell* 30 71–85. 10.1016/j.devcel.2014.06.001 25026034 PMC4109719

[B129] Maes H. Kuchnio A. Peric A. Moens S. Nys K. De Bock K. (2014). Tumor vessel normalization by chloroquine independent of autophagy. *Cancer Cell* 26 190–206. 10.1016/j.ccr.2014.06.025 25117709

[B130] Malhotra R. Warne J. P. Salas E. Xu A. W. Debnath J. (2015). Loss of Atg12, but not Atg5, in pro-opiomelanocortin neurons exacerbates diet-induced obesity. *Autophagy* 11 145–154. 10.1080/15548627.2014.998917 25585051 PMC4502780

[B131] Mammucari C. Milan G. Romanello V. Masiero E. Rudolf R. Del Piccolo P. (2007). FoxO3 controls autophagy in skeletal muscle in vivo. *Cell Metab.* 6 458–471. 10.1016/j.cmet.2007.11.001 18054315

[B132] Marcu R. Choi Y. J. Xue J. Fortin C. L. Wang Y. L. Nagao R. J. (2018). Human Organ-Specific Endothelial Cell Heterogeneity. *Iscience* 4 20–35. 10.1016/j.isci.2018.05.003 30240741 PMC6147238

[B133] Martin J. D. Seano G. Jain R. K. (2019). Normalizing function of tumor vessels: progress. Opportunities, and Challenges. *Annu. Rev. Physiol.* 81 505–534. 10.1146/annurev-physiol-020518-114700 30742782 PMC6571025

[B134] Martinez-Martin N. Maldonado P. Gasparrini F. Frederico B. Aggarwal S. Gaya M. (2017). A switch from canonical to noncanonical autophagy shapes B cell responses. *Science* 355 641–647. 10.1126/science.aal3908 28183981 PMC5805088

[B135] Masiero E. Agatea L. Mammucari C. Blaauw B. Loro E. Komatsu M. (2009). Autophagy is required to maintain muscle mass. *Cell Metab.* 10 507–515. 10.1016/j.cmet.2009.10.008 19945408

[B136] Matta S. K. Kumar D. (2015). AKT mediated glycolytic shift regulates autophagy in classically activated macrophages. *Int. J. Biochem. Cell Biol.* 66 121–133. 10.1016/j.biocel.2015.07.010 26222186

[B137] McGrath M. J. Eramo M. J. Gurung R. Sriratana A. Gehrig S. M. Lynch G. S. (2020). Defective lysosome reformation during autophagy causes skeletal muscle disease. *J. Clin. Invest.* 135124 10.1172/JCI135124 [Epub ahead of print]. PMC7773396 33119550

[B138] Mehrpour M. Esclatine A. Beau I. Codogno P. (2010). Overview of macroautophagy regulation in mammalian cells. *Cell Res.* 20 748–762. 10.1038/cr.2010.82 20548331

[B139] Melendez A. Talloczy Z. Seaman M. Eskelinen E. L. Hall D. H. Levine B. (2003). Autophagy genes are essential for dauer development and life-span extension in *C-elegans*. *Science* 301 1387–1391. 10.1126/science.1087782 12958363

[B140] Mendelson A. Frenette P. S. (2014). Hematopoietic stem cell niche maintenance during homeostasis and regeneration. *Nat. Med.* 20 833–846. 10.1038/nm.3647 25100529 PMC4459580

[B141] Menzies F. M. Fleming A. Caricasole A. Bento C. F. Andrews S. P. Ashkenazi A. (2017). Autophagy and neurodegeneration: pathogenic mechanisms and therapeutic opportunities. *Neuron* 93 1015–1034. 10.1016/j.neuron.2017.01.022 28279350

[B142] Metti S. Gambarotto L. Chrisam M. Baraldo M. Braghetta P. Blaauw B. (2020). The polyphenol pterostilbene ameliorates the myopathic phenotype of collagen VI deficient mice via autophagy induction. *Front. Cell Dev. Biol.* 8:580933. 10.3389/fcell.2020.580933 33134297 PMC7550465

[B143] Miao C. Lei M. Hu W. Han S. Wang Q. (2017). A brief review: the therapeutic potential of bone marrow mesenchymal stem cells in myocardial infarction. *Stem Cell Res. Ther.* 8:242. 10.1186/s13287-017-0697-9 29096705 PMC5667518

[B144] Millecamps S. Julien J. P. (2013). Axonal transport deficits and neurodegenerative diseases. *Nat. Rev. Neurosci.* 14 161–176. 10.1038/nrn3380 23361386

[B145] Mizushima N. (2007). Autophagy: process and function. *Genes Dev.* 21 2861–2873. 10.1101/gad.1599207 18006683

[B146] Mizushima N. Komatsu M. (2011). Autophagy: renovation of cells and tissues. *Cell* 147 728–741. 10.1016/j.cell.2011.10.026 22078875

[B147] Mizushima N. Levine B. (2010). Autophagy in mammalian development and differentiation. *Nat. Cell Biol.* 12 823–830. 10.1038/Ncb0910-823 20811354 PMC3127249

[B148] Mizushima N. Levine B. Cuervo A. M. Klionsky D. J. (2008). Autophagy fights disease through cellular self-digestion. *Nature* 451 1069–1075. 10.1038/nature06639 18305538 PMC2670399

[B149] Mizushima N. Yamamoto A. Matsui M. Yoshimori T. Ohsumi Y. (2004). In vivo analysis of autophagy in response to nutrient starvation using transgenic mice expressing a fluorescent autophagosome marker. *Mol. Biol. Cell* 15 1101–1111. 10.1091/mbc.e03-09-0704 14699058 PMC363084

[B150] Mofarrahi M. Guo Y. Haspel J. A. Choi A. M. Davis E. C. Gouspillou G. (2013). Autophagic flux and oxidative capacity of skeletal muscles during acute starvation. *Autophagy* 9 1604–1620. 10.4161/auto.25955 23955121

[B151] Mortimore G. E. Schworer C. M. (1977). Induction of autophagy by amino-acid deprivation in perfused rat liver. *Nature* 270 174–176. 10.1038/270174a0 927529

[B152] Munz C. (2009). Enhancing immunity through autophagy. *Annu. Rev. Immunol.* 27 423–449. 10.1146/annurev.immunol.021908.132537 19105657

[B153] Murray P. J. Allen J. E. Biswas S. K. Fisher E. A. Gilroy D. W. Goerdt S. (2014). Macrophage activation and polarization: nomenclature and experimental guidelines. *Immunity* 41 14–20. 10.1016/j.immuni.2014.06.008 25035950 PMC4123412

[B154] Nakahira K. Haspel J. A. Rathinam V. A. Lee S. J. Dolinay T. Lam H. C. (2011). Autophagy proteins regulate innate immune responses by inhibiting the release of mitochondrial DNA mediated by the NALP3 inflammasome. *Nat. Immunol.* 12 222–230. 10.1038/ni.1980 21151103 PMC3079381

[B155] Napoli E. Song G. Panoutsopoulos A. Riyadh M. A. Kaushik G. Halmai J. (2018). Beyond autophagy: a novel role for autism-linked Wdfy3 in brain mitophagy. *Sci. Rep.* 8:11348. 10.1038/s41598-018-29421-7 30054502 PMC6063930

[B156] Negrete-Hurtado A. Overhoff M. Bera S. De Bruyckere E. Schatzmuller K. Kye M. J. (2020). Autophagy lipidation machinery regulates axonal microtubule dynamics but is dispensable for survival of mammalian neurons. *Nat. Commun.* 11:1535. 10.1038/s41467-020-15287-9 32210230 PMC7093409

[B157] Nemazanyy I. Blaauw B. Paolini C. Caillaud C. Protasi F. Mueller A. (2013). Defects of Vps15 in skeletal muscles lead to autophagic vacuolar myopathy and lysosomal disease. *EMBO Mol. Med.* 5 870–890. 10.1002/emmm.201202057 23630012 PMC3779449

[B158] Nichenko A. S. Southern W. M. Atuan M. Luan J. Peissig K. B. Foltz S. J. (2016). Mitochondrial maintenance via autophagy contributes to functional skeletal muscle regeneration and remodeling. *Am. J. Physiol. Cell Physiol.* 311 C190–C200. 10.1152/ajpcell.00066.2016 27281480

[B159] Nishiyama J. Miura E. Mizushima N. Watanabe M. Yuzaki M. (2007). Aberrant membranes and double-membrane structures accumulate in the axons of Atg5-null Purkinje cells before neuronal death. *Autophagy* 3 591–596. 10.4161/auto.4964 17912025

[B160] Niven J. Madelon N. Page N. Caruso A. Harle G. Lemeille S. (2019). Macroautophagy in dendritic cells controls the homeostasis and stability of regulatory T Cells. *Cell Rep.* 28 21.e6–29.e26. 10.1016/j.celrep.2019.05.110 31269441

[B161] Nixon R. A. Wegiel J. Kumar A. Yu W. H. Peterhoff C. Cataldo A. (2005). Extensive involvement of autophagy in Alzheimer disease: an immuno-electron microscopy study. *J. Neuropathol. Exp. Neurol.* 64 113–122. 10.1093/jnen/64.2.113 15751225

[B162] Obba S. Hizir Z. Boyer L. Selimoglu-Buet D. Pfeifer A. Michel G. (2015). The PRKAA1/AMPKalpha1 pathway triggers autophagy during CSF1-induced human monocyte differentiation and is a potential target in CMML. *Autophagy* 11 1114–1129. 10.1080/15548627.2015.1034406 26029847 PMC4590592

[B163] Ohsumi Y. (2001). Molecular dissection of autophagy: two ubiquitin-like systems. *Nat. Rev. Mol. Cell Biol.* 2 211–216. 10.1038/35056522 11265251

[B164] Okerlund N. D. Schneider K. Leal-Ortiz S. Montenegro-Venegas C. Kim S. A. Garner L. C. (2017). Bassoon controls presynaptic autophagy through Atg5. *Neuron* 93 897.e7–913.e7. 10.1016/j.neuron.2017.01.026 28231469

[B165] Omilusik K. D. Goldrath A. W. (2017). The origins of memory T cells. *Nature* 552 337–339. 10.1038/d41586-017-08280-8 32080617

[B166] Paludan C. Schmid D. Landthaler M. Vockerodt M. Kube D. Tuschl T. (2005). Endogenous MHC class II processing of a viral nuclear antigen after autophagy. *Science* 307 593–596. 10.1126/science.1104904 15591165

[B167] Paolini A. Omairi S. Mitchell R. Vaughan D. Matsakas A. Vaiyapuri S. (2018). Attenuation of autophagy impacts on muscle fibre development, starvation induced stress and fibre regeneration following acute injury. *Sci. Rep.* 8:9062. 10.1038/s41598-018-27429-7 29899362 PMC5998118

[B168] Park S. K. La Salle D. T. Cerbie J. Cho J. M. Bledsoe A. Nelson A. (2019). Elevated arterial shear rate increases indexes of endothelial cell autophagy and nitric oxide synthase activation in humans. *Am. J. Physiol. Heart Circ. Physiol.* 316 H106–H112. 10.1152/ajpheart.00561.2018 30412436 PMC6734082

[B169] Patente T. A. Pinho M. P. Oliveira A. A. Evangelista G. C. M. Bergami-Santos P. C. Barbuto J. A. M. (2018). Human dendritic cells: their heterogeneity and clinical application potential in cancer immunotherapy. *Front. Immunol.* 9:3176. 10.3389/fimmu.2018.03176 30719026 PMC6348254

[B170] Pengo N. Scolari M. Oliva L. Milan E. Mainoldi F. Raimondi A. (2013). Plasma cells require autophagy for sustainable immunoglobulin production. *Nat. Immunol.* 14 298–305. 10.1038/ni.2524 23354484

[B171] Pua H. H. Dzhagalov I. Chuck M. Mizushima N. He Y. W. (2007). A critical role for the autophagy gene Atg5 in T cell survival and proliferation. *J. Exp. Med.* 204 25–31. 10.1084/jem.20061303 17190837 PMC2118420

[B172] Pua H. H. Guo J. Komatsu M. He Y. W. (2009). Autophagy is essential for mitochondrial clearance in mature T lymphocytes. *J. Immunol.* 182 4046–4055. 10.4049/jimmunol.0801143 19299702

[B173] Qadura M. Terenzi D. C. Verma S. Al-Omran M. Hess D. A. (2018). Concise review: cell therapy for critical limb ischemia: an integrated review of preclinical and clinical studies. *Stem Cells* 36 161–171. 10.1002/stem.2751 29226477

[B174] Raben N. Hill V. Shea L. Takikita S. Baum R. Mizushima N. (2008). Suppression of autophagy in skeletal muscle uncovers the accumulation of ubiquitinated proteins and their potential role in muscle damage in Pompe disease. *Hum. Mol. Genet.* 17 3897–3908. 10.1093/hmg/ddn292 18782848 PMC2638578

[B175] Ramos F. J. Chen S. C. Garelick M. G. Dai D. F. Liao C. Y. Schreiber K. H. (2012). Rapamycin reverses elevated mTORC1 signaling in lamin A/C-deficient mice, rescues cardiac and skeletal muscle function, and extends survival. *Sci. Transl. Med.* 4:144ra103. 10.1126/scitranslmed.3003802 22837538 PMC3613228

[B176] Rastaldo R. Vitale E. Giachino C. (2020). Dual role of autophagy in regulation of mesenchymal stem cell senescence. *Front. Cell Dev. Biol.* 8:276. 10.3389/Fcell.2020.00276 32391362 PMC7193103

[B177] Rathmell J. C. (2012). Metabolism and autophagy in the immune system: immunometabolism comes of age. *Immunol. Rev.* 249 5–13. 10.1111/j.1600-065X.2012.01158.x 22889211 PMC3576876

[B178] Rodriguez-Pinto D. (2005). B cells as antigen presenting cells. *Cell Immunol.* 238 67–75. 10.1016/j.cellimm.2006.02.005 16574086

[B179] Romanelli D. Casartelli M. Cappellozza S. de Eguileor M. Tettamanti G. (2016). Roles and regulation of autophagy and apoptosis in the remodelling of the lepidopteran midgut epithelium during metamorphosis. *Sci. Rep.* 6:32939. 10.1038/Srep32939 27609527 PMC5016986

[B180] Saccone V. Palmieri M. Passamano L. Piluso G. Meroni G. Politano L. (2008). Mutations that impair interaction properties of TRIM32 associated with limb-girdle muscular dystrophy 2H. *Hum. Mutat.* 29 240–247. 10.1002/humu.20633 17994549

[B181] Saera-Vila A. Kish P. E. Louie K. W. Grzegorski S. J. Klionsky D. J. Kahana A. (2016). Autophagy regulates cytoplasmic remodeling during cell reprogramming in a zebrafish model of muscle regeneration. *Autophagy* 12 1864–1875. 10.1080/15548627.2016.1207015 27467399 PMC5066936

[B182] Saitoh T. Fujita N. Jang M. H. Uematsu S. Yang B. G. Satoh T. (2008). Loss of the autophagy protein Atg16L1 enhances endotoxin-induced IL-1beta production. *Nature* 456 264–268. 10.1038/nature07383 18849965

[B183] Sanjurjo L. Aran G. Tellez E. Amezaga N. Armengol C. Lopez D. (2018). CD5L Promotes M2 macrophage polarization through autophagy-mediated upregulation of ID3. *Front. Immunol.* 9:480. 10.3389/fimmu.2018.00480 29593730 PMC5858086

[B184] Sarkar S. Floto R. A. Berger Z. Imarisio S. Cordenier A. Pasco M. (2005). Lithium induces autophagy by inhibiting inositol monophosphatase. *J. Cell Biol*, 170 1101–1111. 10.1083/jcb.200504035 16186256 PMC2171537

[B185] Sarparanta J. Jonson P. H. Golzio C. Sandell S. Luque H. Screen M. (2012). Mutations affecting the cytoplasmic functions of the co-chaperone DNAJB6 cause limb-girdle muscular dystrophy. *Nat. Genet.* 44 S451–S452. 10.1038/ng.1103 22366786 PMC3315599

[B186] Sbrana F. V. Cortini M. Avnet S. Perut F. Columbaro M. De Milito A. (2016). The role of autophagy in the maintenance of stemness and differentiation of mesenchymal stem cells. *Stem Cell Rev. Rep.* 12 621–633. 10.1007/s12015-016-9690-4 27696271

[B187] Schaaf M. B. Houbaert D. Mece O. Agostinis P. (2019). Autophagy in endothelial cells and tumor angiogenesis. *Cell Death Differ.* 26 665–679. 10.1038/s41418-019-0287-8 30692642 PMC6460396

[B188] Segales J. Perdiguero E. Serrano A. L. Sousa-Victor P. Ortet L. Jardi M. (2020). Sestrin prevents atrophy of disused and aging muscles by integrating anabolic and catabolic signals. *Nat. Commun.* 11:189. 10.1038/s41467-019-13832-9 31929511 PMC6955241

[B189] Simonsen A. Tooze S. A. (2009). Coordination of membrane events during autophagy by multiple class III PI3-kinase complexes. *J. Cell Biol.* 186 773–782. 10.1083/jcb.200907014 19797076 PMC2753151

[B190] Skobo T. Benato F. Grumati P. Meneghetti G. Cianfanelli V. Castagnaro S. (2014). Zebrafish ambra1a and ambra1b knockdown impairs skeletal muscle development. *PLoS One* 9:e99210. 10.1371/journal.pone.0099210 24922546 PMC4055674

[B191] Smith J. A. (2018). Regulation of cytokine production by the unfolded protein response. Implications for infection and autoimmunity. *Front. Immunol.* 9:422. 10.3389/fimmu.2018.00422 29556237 PMC5844972

[B192] Son J. H. Shim J. H. Kim K. H. Ha J. Y. Han J. Y. (2012). Neuronal autophagy and neurodegenerative diseases. *Exp. Mol. Med.* 44 89–98. 10.3858/emm.2012.44.2.031 22257884 PMC3296817

[B193] Song C. Song C. Tong F. (2014). Autophagy induction is a survival response against oxidative stress in bone marrow-derived mesenchymal stromal cells. *Cytotherapy* 16 1361–1370. 10.1016/j.jcyt.2014.04.006 24980657

[B194] Song K. Y. Guo X. M. Wang H. Q. Zhang L. Huang S. Y. Huo Y. C. (2020). MBNL1 reverses the proliferation defect of skeletal muscle satellite cells in myotonic dystrophy type 1 by inhibiting autophagy via the mTOR pathway. *Cell Death Dis.* 11:545. 10.1038/s41419-020-02756-8 32683410 PMC7368861

[B195] Sou Y. S. Waguri S. Iwata J. Ueno T. Fujimura T. Hara T. (2008). The Atg8 conjugation system is indispensable for proper development of autophagic isolation membranes in mice. *Mol. Biol. Cell* 19 4762–4775. 10.1091/mbc.e08-03-0309 18768753 PMC2575156

[B196] Spampanato C. Feeney E. Li L. Cardone M. Lim J. A. Annunziata F. (2013). Transcription factor EB (TFEB) is a new therapeutic target for Pompe disease. *EMBO Mol. Med.* 5 691–706. 10.1002/emmm.201202176 23606558 PMC3662313

[B197] Spengler K. Kryeziu N. Grosse S. Mosig A. S. Heller R. (2020). VEGF triggers transient induction of autophagy in endothelial cells via AMPK alpha 1. *Cells* 9:687. 10.3390/Cells9030687 32168879 PMC7140637

[B198] Sprott D. Poitz D. M. Korovina I. Ziogas A. Phieler J. Chatzigeorgiou A. (2019). Endothelial-specific deficiency of ATG5 (Autophagy Protein 5) attenuates ischemia-related angiogenesis. *Arteriosc. Thromb. Vasc. Biol.* 39 1137–1148. 10.1161/ATVBAHA.119.309973 31070476

[B199] Sweeney M. Foldes G. (2018). It takes two: endothelial-perivascular cell cross-talk in vascular development and disease. *Front. Cardiovasc. Med.* 5:154. 10.3389/fcvm.2018.00154 30425990 PMC6218412

[B200] Tang A. H. Rando T. A. (2014). Induction of autophagy supports the bioenergetic demands of quiescent muscle stem cell activation. *EMBO J.* 33 2782–2797. 10.15252/embj.201488278 25316028 PMC4282556

[B201] Tang G. Gudsnuk K. Kuo S. H. Cotrina M. L. Rosoklija G. Sosunov A. (2014). Loss of mTOR-dependent macroautophagy causes autistic-like synaptic pruning deficits. *Neuron* 83 1131–1143. 10.1016/j.neuron.2014.0 25155956 PMC4159743

[B202] Torisu K. Singh K. K. Torisu T. Lovren F. Liu J. Pan Y. (2016). Intact endothelial autophagy is required to maintain vascular lipid homeostasis. *Aging Cell* 15 187–191. 10.1111/acel.12423 26780888 PMC4717267

[B203] Tsukada M. Ohsumi Y. (1993). Isolation and characterization of autophagy-defective mutants of saccharomyces-cerevisiae. *Febs Lett.* 333 169–174. 10.1016/0014-5793(93)80398-E 8224160

[B204] Valecka J. Almeida C. R. Su B. Pierre P. Gatti E. (2018). Autophagy and MHC-restricted antigen presentation. *Mol. Immunol.* 99 163–170. 10.1016/j.molimm.2018.05.009 29787980

[B205] Vanhauwaert R. Kuenen S. Masius R. Bademosi A. Manetsberger J. Schoovaerts N. (2017). The SAC1 domain in synaptojanin is required for autophagosome maturation at presynaptic terminals. *EMBO J.* 36 1392–1411. 10.15252/embj.201695773 28331029 PMC5430236

[B206] Vergadi E. Ieronymaki E. Lyroni K. Vaporidi K. Tsatsanis C. (2017). akt signaling pathway in macrophage activation and M1/M2 polarization. *J. Immunol.* 198 1006–1014. 10.4049/jimmunol.1601515 28115590

[B207] Vignali D. A. Collison L. W. Workman C. J. (2008). How regulatory T cells work. *Nat. Rev. Immunol.* 8 523–532. 10.1038/nri2343 18566595 PMC2665249

[B208] Vion A. C. Kheloufi M. Hammoutene A. Poisson J. Lasselin J. Devue C. (2017). Autophagy is required for endothelial cell alignment and atheroprotection under physiological blood flow. *Proc. Natl. Acad. Sci. U.S.A.* 114 E8675–E8684. 10.1073/pnas.1702223114 28973855 PMC5642679

[B209] Wei J. Long L. Yang K. Guy C. Shrestha S. Chen Z. (2016). Autophagy enforces functional integrity of regulatory T cells by coupling environmental cues and metabolic homeostasis. *Nat. Immunol.* 17 277–285. 10.1038/ni.3365 26808230 PMC4755832

[B210] Wen Y. T. Zhang J. R. Kapupara K. Tsai R. K. (2019). mTORC2 activation protects retinal ganglion cells via Akt signaling after autophagy induction in traumatic optic nerve injury. *Exp. Mol. Med.* 51 1–11. 10.1038/s12276-019-0298-z 31409770 PMC6802655

[B211] Willinger T. Flavell R. A. (2012). Canonical autophagy dependent on the class III phosphoinositide-3 kinase Vps34 is required for naive T-cell homeostasis. *Proc. Natl. Acad. Sci. U.S.A.* 109 8670–8675. 10.1073/pnas.1205305109 22592798 PMC3365213

[B212] Wu X. Fleming A. Ricketts T. Pavel M. Virgin H. Menzies F. M. (2016). Autophagy regulates Notch degradation and modulates stem cell development and neurogenesis. *Nat. Commun.* 7:10533. 10.1038/ncomms10533 26837467 PMC4742842

[B213] Wu X. Won H. Rubinsztein D. C. (2013). Autophagy and mammalian development. *Biochem. Soc. Trans*, 41 1489–1494. 10.1042/BST20130185 24256242

[B214] Yamamoto A. Yue Z. (2014). Autophagy and its normal and pathogenic states in the brain. *Annu. Rev. Neurosci.* 37 55–78. 10.1146/annurev-neuro-071013-014149 24821313

[B215] Yan Y. Finkel T. (2017). Autophagy as a regulator of cardiovascular redox homeostasis. *Free Radic. Biol. Med.* 109 108–113. 10.1016/j.freeradbiomed.2016.12.003 27940349 PMC5462893

[B216] Yoshii S. R. Kuma A. Akashi T. Hara T. Yamamoto A. Kurikawa Y. (2016). Systemic analysis of Atg5-null mice rescued from neonatal lethality by transgenic ATG5 expression in neurons. *Dev. Cell* 39 116–130. 10.1016/j.devcel.2016.09.001 27693508

[B217] Zecchini S. Giovarelli M. Perrotta C. Morisi F. Touvier T. Di Renzo I. (2019). Autophagy controls neonatal myogenesis by regulating the GH-IGF1 system through a NFE2L2- and DDIT3-mediated mechanism. *Autophagy* 15 58–77. 10.1080/15548627.2018.1507439 30081710 PMC6287695

[B218] Zeng N. D’Souza R. F. Mitchell C. J. Cameron-Smith D. (2018). Sestrins are differentially expressed with age in the skeletal muscle of men: a cross-sectional analysis. *Exp. Gerontol.* 110 23–34. 10.1016/j.exger.2018.05.006 29751091

[B219] Zhang D. Y. Chen Y. F. Xu X. B. Xiang H. Y. Shi Y. Z. Gao Y. (2020). Autophagy inhibits the mesenchymal stem cell aging induced by D-galactose through ROS/JNK/p38 signalling. *Clin. Exp. Pharmacol. Physiol.* 47 466–477. 10.1111/1440-1681.13207 31675454

[B220] Zhang J. Chen L. Xiong F. Zhang S. Huang K. Zhang Z. (2019). Autophagy in regulatory T cells: a double-edged sword in disease settings. *Mol. Immunol.* 109 43–50. 10.1016/j.molimm.2019.02.004 30852245

[B221] Zhang Q. Yang Y. J. Wang H. Dong Q. T. Wang T. J. Qian H. Y. (2012). Autophagy activation: a novel mechanism of atorvastatin to protect mesenchymal stem cells from hypoxia and serum deprivation via AMP-activated protein kinase/mammalian target of rapamycin pathway. *Stem Cells Dev.* 21 1321–1332. 10.1089/scd.2011.0684 22356678 PMC3353754

[B222] Zhang Y. Morgan M. J. Chen K. Choksi S. Liu Z. G. (2012). Induction of autophagy is essential for monocyte-macrophage differentiation. *Blood* 119 2895–2905. 10.1182/blood-2011-08-372383 22223827 PMC3327464

[B223] Zhao X. C. Nedvetsky P. Stanchi F. Vion A. C. Popp O. Zuhlke K. (2019). Endothelial PKA activity regulates angiogenesis by limiting autophagy through phosphorylation of ATG16L1. *eLife* 8:e46380. 10.7554/eLife.46380 31580256 PMC6797479

[B224] Zhuang S. F. Liu D. X. Wang H. J. Zhang S. H. Wei J. Y. Fang W. G. (2017). Atg7 regulates brain angiogenesis via NF-kappaB-Dependent IL-6 production. *Int. J. Mol. Sci.s* 18:968. 10.3390/ijms18050968 28467355 PMC5454881

